# Message-Based vs Video-Based Psychotherapy for Depression: A Randomized Clinical Trial

**DOI:** 10.1001/jamanetworkopen.2025.40065

**Published:** 2025-10-30

**Authors:** Michael D. Pullmann, Julien Rouvere, Patrick J. Raue, Isabell R. Griffith Fillipo, Brittany A. Mosser, Patrick J. Heagerty, Nicole Fridling-Cook, Aarthi Padmanabhan, Thomas D. Hull, Patricia A. Areán

**Affiliations:** 1 Department of Psychiatry and Behavioral Sciences, School of Medicine, University of Washington, Seattle; 2 Department of Biostatistics, School of Public Health, University of Washington, Seattle; 3 Talkspace Inc, New York, New York

## Abstract

**Importance:**

There is a need to determine the relative effect of message-based psychotherapy (MBP), an asynchronous approach that uses emails, texts, or voice or video messages to permit therapeutic exchanges, compared with video-based psychotherapy (VBP) and whether a combination of modalities would result in better outcomes for those who do not respond to either treatment alone.

**Objectives:**

To compare MBP with VBP on a commercial digital mental health platform and test combinations of modalities for participants who did not respond to single-modality treatment.

**Design, Setting, and Participants:**

In this sequential multiple assignment randomized clinical trial, psychotherapy was delivered by therapists on a commercial digital mental health platform from January 10, 2022, to January 14, 2024, among 850 participants who were 18 years of age or older, English or Spanish speaking, living in the US in a state where the digital mental health platform had available therapists, scored 10 or more on the 9-item Patient Health Questionnaire (PHQ-9), and received a diagnosis of depression during intake assessment.

**Interventions:**

At baseline, participants were randomized to MBP or weekly VBP. At week 6, nonresponders were rerandomized to MBP with weekly or monthly VBP. Participants received treatment for 12 weeks.

**Main Outcomes and Measures:**

Primary outcomes included depression severity (measured by the PHQ-9), social functioning, response to treatment, and remission. Secondary outcomes included treatment engagement, therapeutic alliance, and indicators of treatment quality and satisfaction. Analysis was performed on an intention-to-treat basis.

**Results:**

The analytic sample included 850 participants (mean [SD] age, 33.8 [10.5] years; 562 women [66.1%]; mean [SD] PHQ-9 score, 15.0 [4.8]), with 423 randomized to MBP and 427 to VBP. Treatment disengagement by week 5 was more likely for VBP than MBP (VBP, 91 [21.3%]; MBP, 56 [13.2%]; Cramér *V* = 0.10; 95% CI, 0.03-0.13; *P* = .003). There were no significant differences on depression or social functioning score changes between MBP and VBP or on depression score changes for nonresponders randomized to MBP with weekly vs monthly VBP. At week 12, MBP and VBP did not differ in the proportion of participants who responded to treatment (MBP, 144 of 303 [47.5%]; VBP, 134 of 284 [47.2%]; Cramér *V* < .001; 95% CI, −0.08 to 0.09; *P* = .99) or who experienced remission (MBP, 95 of 303 [31.4%]; VBP, 86 of 284 [30.3%]; Cramér *V* = 0.01; 95% CI, −0.07 to 0.09; *P* = .85). Among nonresponders, VBP had a stronger initial therapeutic alliance than MBP at week 4 (*P* < .001; *d* = 0.48-0.57). Among participants assessed for rerandomization, there were no statistically significant differences among those who responded to treatment by week 5 (MBP, 105 of 363 [28.9%]; VBP, 93 of 336 [27.7%]; Cramér *V* = 0.01; 95% CI, −0.06 to 0.08; *P* = .78). Therapeutic alliance ratings increased across all conditions by week 10; however, these changes were not statistically significant. Video-based psychotherapy was more frequently recommended than MBP (VBP, 69 of 71 [97.2%]; MBP, 70 of 80 [87.5%]; odds ratio, 0.18; 95% CI, 0.04-0.88; *P* = .03). There were no significant differences in clinical outcomes between nonresponders randomized to weekly vs monthly VBP.

**Conclusions and Relevance:**

In this sequential multiple assignment randomized clinical trial comparing MBP with VBP, there were no differences between groups on improvement in depression or social functioning. More participants in the VBP group disengaged from treatment, while VBP also had greater therapeutic alliance early in treatment among nonresponders. There were no differential effects from rerandomizing nonresponders. Findings reinforced MBP as a viable alternative to VBP. Broader insurance reimbursement for MBP could improve access to evidence-based care. Future research should explore optimizing early alliance-building in MBP.

**Trial Registration:**

ClinicalTrials.gov Identifier: NCT04513080

## Key Points

**Question:**

Is message-based psychotherapy (MBP) more effective than video-based psychotherapy (VBP) in improving clinical outcomes among patients with depression?

**Findings:**

This sequential multiple assignment randomized clinical trial including 850 adults found similar outcomes between MBP and VBP, contradicting the hypothesis that MBP would be superior due to its immediacy. Among participants who were evaluated for level 2 randomization (363 received MBP and 336 received VBP), there were no statistically significant differences in those who responded to treatment by week 5 (28.9% of those who received MBP vs 27.7% of those who received VBP).

**Meaning:**

This study found that MBP was a viable alternative to VBP; insurance reimbursement for MBP could improve access to evidence-based care.

## Teaser

This randomized clinical trial compares message-based psychotherapy with video-based psychotherapy on a commercial digital mental health platform and tests combinations of modalities for participants who did not respond to single-modality treatment.

## Introduction

Depression stands as the foremost cause of disability and mortality worldwide, correlating with heightened suicide risk, diminished school and work performance, compromised quality of life, and significant economic burden.^1,2,3^ Without treatment, only 23% of adults with major depression remit within 3 months.^4^

Psychotherapies such as cognitive behavioral therapy have a strong evidence base for treating mental health conditions. They are often preferred by individuals seeking care for depression,^5^ yet most who are able to access psychotherapy do not receive a full course of treatment.^6,7,8,9,10,11^ Video-based psychotherapy (VBP) has emerged as an engaging and accessible alternative and has comparable efficacy with traditional face-to-face therapy across diverse demographics.^12,13,14^ However, VBP still constrains patients to similar time barriers as conventional psychotherapy.

Message-based psychotherapy (MBP) is a novel approach that uses emails, texts, or voice or video messages to permit therapeutic exchanges. Its asynchronous nature allows for accessible and flexible patient-therapist interaction during moments of greatest need, facilitates more frequent contact (which is associated with steeper recovery curves), and creates a record of interactions that patients can review for deeper integration and action plan implementation.^15,16,17^ Although several digital mental health companies offer message-based care, few studies on the effectiveness of message-based care have been published. Data are mixed on the effectiveness of MBP; studies have largely been conducted using automated text messages^18,19^ or in combination with traditional or other modes of psychotherapy.^20,21,22,23,24,25,26^ Small-sample research with a commercial digital mental health platform^27^ found that therapy using MBP was associated with clinically significant treatment remission,^28^ lower costs, greatly reduced wait times, and high satisfaction on affordability, convenience, and effectiveness.^29^

Given the limited data to support MBP as a mode of therapy delivery and the established evidence base for VBP, the present study aimed to determine the effect of MBP compared with VBP as well as the relative effectiveness and optimal treatment sequence for combined modalities. We report data collected during the second phase of a National Institutes of Mental Health–funded Small Business Innovations Research Award to conduct a large, remote, 2-phase sequential multiple assignment randomized clinical trial^30^ in a partnership between a digital mental health platform and the University of Washington. A pilot of the present study, conducted with 215 participants randomly assigned to MBP or VBP, found no differences in rates of change between conditions on depression, anxiety, disability, and global improvement ratings across 12 weeks, as well as no adverse events.^31^

Due to MBP’s flexibility, its ability to meet an in-the-moment need, frequency of patient-therapist contact, and record of interactions to support action plan implementation, we hypothesized superiority of MBP over VBP on treatment of depression. Specifically, we hypothesized that (1) individuals randomized to MBP would have greater improvements than those randomized to VBP on depression and social functioning, and would be more likely to respond and experience remission at 5 and 12 weeks of care; (2) for nonresponders, those rerandomized to receive MBP plus weekly VBP would have greater improvements in depression and be more likely to respond to treatment than those randomized to MBP plus monthly VBP, based on data supporting the benefits of more frequent meetings^15^; and (3) individuals with greater baseline anxiety would have better response to MBP vs VBP at 5 and 12 weeks. Furthermore, we explored whether there were differences in therapeutic alliance and indicators of treatment quality and satisfaction.

## Methods

This pragmatic sequential multiple assignment randomized clinical trial follows the Consolidated Standards of Reporting Trials (CONSORT) reporting guideline. A pragmatic trial is the study design for an effectiveness trial, which distinguishes it from trial designs in an efficacy study; a pragmatic trial has a large sample size and aims for generalizability and external validity.^32^ This study was approved by the University of Washington institutional review board and conducted between January 10, 2022, and January 14, 2024. Participants were recruited on the digital mental health platform, social media platforms, and the Mental Health America website.^33^ Eligible participants self-administered electronic informed consent via REDCap,^34,35^ with 3 comprehension check questions that required correct responses to proceed. The trial protocol and statistical analysis plan are in Supplement 1.

### Participants

Inclusion criteria for participants were age 18 years or older, English or Spanish speaking, living in the US in a state where the digital mental health platform had available clinicians for the study, a score of 10 or more on the 9-item Patient Health Questionnaire (PHQ-9),^36^ and receipt of a diagnosis of depression during intake assessment. Exclusion criteria were active suicidal ideation, diagnosis of psychosis, or self-reported difficulty using message- or video-based care. See eMethods 1 in Supplement 2 for procedures used to manage fraudulent participation.

### Therapists

We recruited 76 therapists with master’s or doctoral level degrees and verified clinical licensures. Therapist training and clinical certification were verified by the digital mental health platform. We did not collect detailed data on therapeutic orientation from therapists, but in another study using 1599 digital mental health platform clinician network therapists, 61.0% reported offering cognitive behavioral therapy, 40.3% reported offering third wave cognitive behavioral interventions such as mindfulness-based approaches, and 25.5% reported offering psychodynamic or relational interventions.^37^ Therapists attended a day-long booster training session on evidence-based psychotherapy for depression and study procedures. Treatment fidelity was not monitored in this effectiveness study.

### Interventions

#### Message-Based Psychotherapy

Asynchronous, as-needed text-based or email-based communication occurred between therapists and participants over a secure platform. Therapists responded 15 minutes to 14 hours after contact.

#### Video-Based Psychotherapy

Therapists provided 30 to 45 minutes of psychotherapy using a secure, Heath Insurance Portability and Accountability Act–compliant video conferencing service.

### Procedures

Participants were screened, consented, met an intake clinician, matched with a therapist, and received up to 12 weeks of treatment. Randomization at baseline to MBP or weekly VBP (ie, first condition; R1) was triggered by a research coordinator on enrollment using simple randomization with prespecified, concealed blocks by therapist to ensure condition balance.

At week 6, participants were rerandomized (ie, second condition; R2). Those who responded to treatment remained in their original condition. Message-based psychotherapy participants who did not improve were randomly assigned to MBP plus supplemental weekly or monthly VBP. Video-based psychotherapy participants who did not improve received additional MBP and were rerandomized to continue weekly or monthly VBP. If the PHQ-9 score was missing from weeks 3 to 5, therapists who reported sufficient interactions with clients provided Clinical Global Impressions–Improvement (CGI-I) scale ratings.^38^ Participants with CGI-I scores of 2 or less (much or very much improved) remained in their original condition. Assessments were distributed via REDCap. See eMethods 2 in Supplement 2 for the data management statement and eMethods 3 in Supplement 2 for intention-to-treat procedures.

### Measures

The primary outcome was measured by the PHQ-9, which assesses depression severity over the last 2 weeks.^36^ Items are rated on a scale from 0 to 3 (range, 0-27), with higher scores indicating greater severity.^39,40^ Primary outcomes also included response to treatment (≥50% reduction in PHQ-9 total scores or CGI-I scores ≤2), remission (PHQ-9 score <5), and social functioning as measured by the 8-item Neuro-Quality of Life, version 1.0 (Neuro-QOL) Ability to Participate in Social Roles and Activities short form.^41^ Neuro-QOL items focus on difficulty participating in social, family, and work activities and are rated on a scale from 1 to 5 (range, 8-40), transformed into *t* scores. Higher scores indicate higher social functioning.

Secondary outcomes included (1) treatment disengagement (switch to nonstudy therapist, withdrawal from study or treatment, or insufficient engagement); (2) therapeutic alliance, measured by the Working Alliance Inventory–Short Revised (WAI-SR)^42^; (3) quality of care in the past 4 weeks, measured by an adapted version of item 28 of the Experience of Care and Health Outcomes (ECHO) survey^43^ on a scale from 0 (worst) to 10 (best), and an item asking how much participants were helped by treatment on a scale from 0 (not at all) to 3 (a lot); and (4) treatment satisfaction measured by 3 yes or no items asking participants if the treatment met their goal, if they planned to continue using treatment, and if they would recommend treatment to others. See eMethods 4 in Supplement 2 for measure descriptions.

Baseline anxiety severity was measured using the 7-item Generalized Anxiety Disorder (GAD-7) scale.^44,45^ At week 5, therapists were asked to rate their client’s improvement since the beginning of treatment with the CGI-I scale.^38^ Participants self-reported demographics at baseline, the PHQ-9 and Neuro-QOL at baseline and weekly for 12 weeks, the WAI-SR at weeks 4 and 10, treatment quality measures at week 10, and treatment satisfaction at weeks 4 and 12. Demographic characteristics were collected to describe the sample. Racial categories included American Indian or Alaska Native, Asian, Black or African American, Native Hawaiian or Other Pacific Islander, White, prefer not to say, and prefer to self-describe (included Hispanic; Jewish; Latina, Latino, or Latinx; Mestizo; Mexican; Mexican American; Mexicana; Middle Eastern or North African; and multiracial). Participants who selected more than 1 racial category were reported as having multiple responses (included Hispanic, Mexican and Irish, and Middle Eastern). Ethnic categories included Hispanic, not Hispanic, and prefer not to say. Race and ethnicity data were collected to provide descriptive data on the sample characteristics.

### Statistical Analysis

Analysis was performed on an intention-to-treat basis. Associations between baseline variables and R1 conditions were analyzed via *t* tests, χ^2^ tests, and Fisher exact tests. Associations between conditions and treatment disengagement by week 5, response to treatment by week 5 and at week 12, and remission by week 5 and at week 12 were analyzed via χ^2^ tests with Cramér *V* effect sizes. Analyses including time as a continuous covariate (eg, PHQ-9 score from baseline to week 5 and from week 6 to week 12, Neuro-QOL score from baseline to week 12) were tested using 3-level mixed effects regression models using full information maximum likelihood (FIML) and an unstructured covariance matrix with time point nested within client nested within therapist. Client-level effects were allowed to randomly vary, clustered by therapist. Effect sizes were computed via the Cohen *d* absolute value (difference between model-estimated mean values divided by pooled baseline SDs). Pooled SDs at week 6 were used in the model estimating PHQ-9 scores by R2. Comparisons of R2 examined estimated means between responders (MBP vs VBP), MBP nonresponders (MBP plus weekly VBP vs MBP plus monthly VBP), and VBP nonresponders (weekly VBP plus MBP vs monthly VBP plus MBP). All *P* values were from 2-sided tests and results were deemed statistically significant at *P* < .05.

Analyses estimating change in WAI-SR scores from weeks 4 to 10 with time and R2 condition as categorical covariates, quality of care at week 10, and amount helped at week 10 were conducted using 2-level linear mixed-effects models using FIML and an unstructured covariance matrix with client nested within therapist. Pooled SDs at week 4 for the WAI-SR and at week 10 for quality of care and amount helped were used for effect sizes. Treatment satisfaction outcomes at week 12 were analyzed via 2-level logistic regression models with Cholesky factorization with client nested within therapist. Post hoc analyses were conducted to explore associations between treatment response and treatment satisfaction outcomes at week 4 via χ^2^ tests. To assess the robustness of our χ^2^ results, we conducted sensitivity analyses using 2-level linear mixed-effects regression models using FIML. These analyses yielded no differences in statistical significance as the original χ^2^ tests. Given the equivalence of results and to ease interpretability for readers, we report χ^2^ results for these analyses.

The Benjamini-Hochberg procedure^46^ adjusted for familywise error within hypotheses using a conservative false discovery rate of 0.05 for comparisons between baseline variables by R1 condition, hypothesis 1, and hypothesis 2 and a more lenient false discovery rate of 0.10 for exploratory hypotheses. We report unadjusted *P* values; the critical false discovery rate–corrected *P* value is provided when significance was affected after correction. Data were processed in R, version 4.3.2 (R Project for Statistical Computing).^47^ Models were analyzed using SAS, version 9.4^48^ PROC MIXED and PROC GLIMMIX (SAS Institute Inc). See eMethods 5 in Supplement 2 for assignment of therapist values for participants who switched therapists.

Total scores were prorated when data were available for at least half of the items. Missingness rates ranged from 15.9% to 24.8% from baseline to week 5 among all participants and 14.1% to 22.3% from weeks 6 to 12 among R2 participants.

A priori power analyses assumed complete data on 900 participants at 6 weeks and 800 participants at 12 weeks, assuming that 40% of participants would not show improvement at 6 weeks. These analyses indicated 80% power to detect a 0.20 SD between MBP and VBP at 6 weeks, and 0.32 SD between nonresponders (weekly vs monthly VBP) when pooling across R1 strata at 12 weeks. These assumptions were very similar to data collection rates.

## Results

### Participant Data

**Figure 1.**
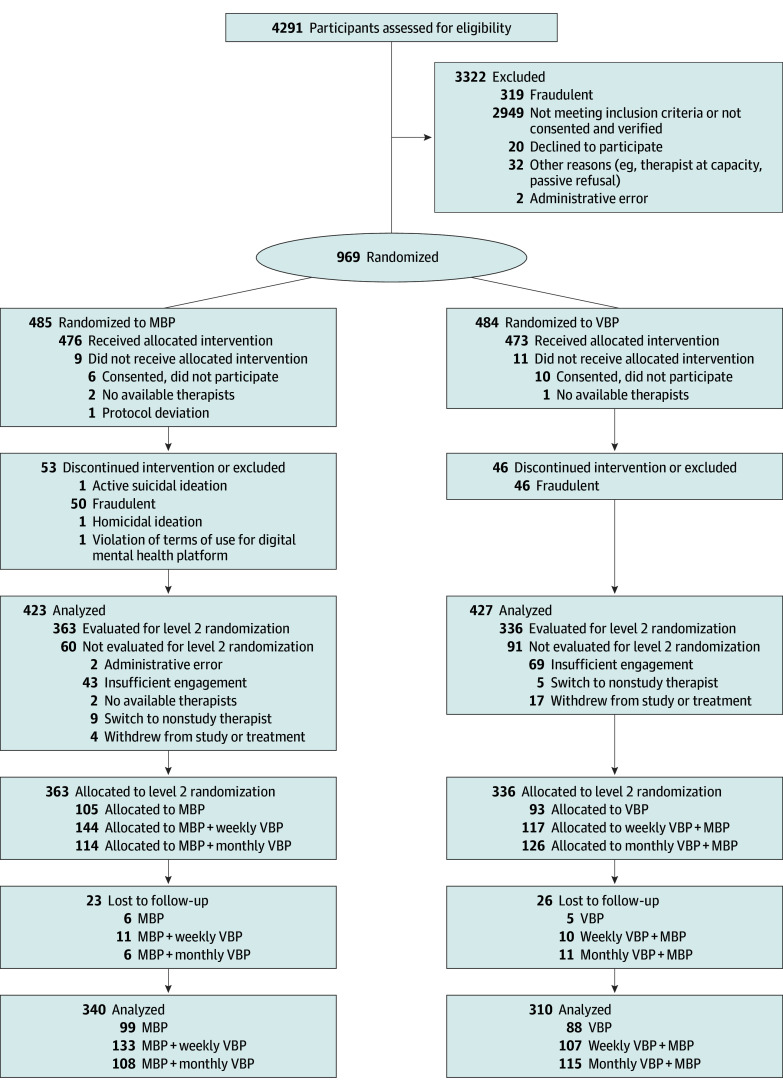
Flow Diagram MBP indicates message-based psychotherapy; MBP + monthly VBP, participants rerandomized from MBP to MBP + monthly VBP; MBP + weekly VBP, participants rerandomized from MBP to MBP + weekly VBP; monthly VBP + MBP, participants rerandomized from weekly VBP to MBP + monthly VBP; weekly VBP + MBP: participants rerandomized from weekly VBP to MBP + weekly VBP; and VBP, video-based psychotherapy.

At baseline, 969 participants were randomized to MBP or VBP (Figure 1). The most common reason for ineligibility after randomization was suspected fraudulent participation (n = 96). The analytic sample comprised 850 individuals (423 received MBP, 427 received VBP; mean [SD] age, 33.8 [10.5] years; 562 women [66.1%], 231 men [27.2%], and 28 nonbinary individuals [3.3%]) (Table 1). The sample included 6 American Indian or Alaska Native participants (0.7%), 45 Asian participants (5.3%), 190 Black or African American participants (22.4%), 120 Hispanic participants (14.1%), 1 Native Hawaiian or Other Pacific Islander participant (0.1%), and 512 White participants (60.2%). There were small percentages who preferred to self-describe race and ethnicity (n = 18 [2.1%]), preferred not to identify race and ethnicity (n = 16 [1.9%]), or who provided multiple races (n = 55 [6.5%]). There were no statistically significant differences in baseline variables between conditions.

**Table 1.** Demographic Characteristics

Characteristic	Participants, No. (%)		
	Total (N = 850)	MBP (n = 423)	VBP (n = 427)
Age, mean (SD) [range], y	33.8 (10.5) [18.5-73.6]	33.4 (10.1) [18.5-73.6]	34.1 (10.9) [18.8-73.6]
Gender			
Male	231 (27.2)	116 (27.4)	115 (26.9)
Female	562 (66.1)	275 (65.0)	287 (67.2)
Transgender male	8 (0.9)	5 (1.2)	3 (0.7)
Transgender female	3 (0.4)	1 (0.2)	2 (0.5)
Genderqueer	6 (0.7)	4 (0.9)	2 (0.5)
Gender variant	1 (0.1)	1 (0.2)	0
Gender other	4 (0.5)	0	4 (0.9)
Nonbinary	28 (3.3)	16 (3.8)	12 (2.8)
Missing	7 (0.8)	5 (1.2)	2 (0.5)
Ethnicity			
Hispanic	120 (14.1)	62 (14.7)	58 (13.6)
Not Hispanic	713 (83.9)	351 (83.0)	362 (84.8)
Prefer not to say	10 (1.2)	5 (1.2)	5 (1.2)
Missing	7 (0.8)	5 (1.2)	2 (0.5)
Race			
American Indian or Alaska Native	6 (0.7)	1 (0.2)	5 (1.2)
Asian	45 (5.3)	24 (5.7)	21 (4.9)
Black or African American	190 (22.4)	97 (22.9)	93 (21.8)
Native Hawaiian or Other Pacific Islander	1 (0.1)	0	1 (0.2)
White	512 (60.2)	259 (61.2)	253 (59.3)
Prefer not to say	16 (1.9)	6 (1.4)	10 (2.3)
Prefer to self-describe^a^	18 (2.1)	9 (2.1)	9 (2.1)
Multiple responses^b^	55 (6.5)	22 (5.2)	33 (7.7)
Missing	7 (0.8)	5 (1.2)	2 (0.5)
Marital status			
Married or partnered	324 (38.1)	174 (41.1)	150 (35.1)
Divorced	45 (5.3)	16 (3.8)	29 (6.8)
Separated	23 (2.7)	14 (3.3)	9 (2.1)
Widowed	9 (1.1)	2 (0.5)	7 (1.6)
Never married	437 (51.4)	209 (49.4)	228 (53.4)
Prefer not to say	5 (0.6)	3 (0.7)	2 (0.5)
Missing	7 (0.8)	5 (1.2)	2 (0.5)
Primary language English			
Yes	822 (96.7)	404 (95.5)	418 (97.9)
No	21 (2.5)	14 (3.3)	7 (1.6)
Missing	7 (0.8)	5 (1.2)	2 (0.5)
Educational level			
Some high school or less than high school diploma	15 (1.8)	4 (0.9)	11 (2.6)
High school diploma or GED certification	83 (9.8)	42 (9.9)	41 (9.6)
Some college	195 (22.9)	91 (21.5)	104 (24.4)
Associate’s degree	77 (9.1)	37 (8.7)	40 (9.4)
Bachelor’s degree	312 (36.7)	158 (37.4)	154 (36.1)
Master’s degree	120 (14.1)	62 (14.7)	58 (13.6)
Professional degree	14 (1.6)	6 (1.4)	8 (1.9)
Doctoral degree	27 (3.2)	18 (4.3)	9 (2.1)
Missing	7 (0.8)	5 (1.2)	2 (0.5)
Employed			
Yes	603 (70.9)	305 (72.1)	298 (69.8)
No	223 (26.2)	104 (24.6)	119 (27.9)
Prefer not to say	17 (2.0)	9 (2.1)	8 (1.9)
Missing	7 (0.8)	5 (1.2)	2 (0.5)
Income, $			
<25 000	264 (31.1)	124 (29.3)	140 (32.8)
25 000-49 999	172 (20.2)	86 (20.3)	86 (20.1)
50 000-74 999	133 (15.6)	70 (16.5)	63 (14.8)
75 000-99 999	105 (12.4)	57 (13.5)	48 (11.2)
≥100 000	128 (15.1)	65 (15.4)	63 (14.8)
Prefer not to say	41 (4.8)	16 (3.8)	25 (5.9)
Missing	7 (0.8)	5 (1.2)	2 (0.5)
First time in therapy			
Yes	386 (45.4)	193 (45.6)	193 (45.2)
No	457 (53.8)	225 (53.2)	232 (54.3)
Missing	7 (0.8)	5 (1.2)	2 (0.5)

Abbreviations: GED, General Educational Development; MBP, message-based psychotherapy; VBP, video-based psychotherapy.

^a^ Write-in responses for “Prefer to self-describe” included Hispanic; Jewish; Latina, Latino, or Latinx; Mestizo; Mexican; Mexican American; Mexicana; Middle Eastern or North African; and multiracial.

^b^ Write-in responses for “Prefer to self-describe” when selected in addition to another response option included Hispanic, Mexican and Irish, and Middle Eastern.

### Response to Treatment, Remission, and Treatment Engagement

Of the analytic sample (n = 850), 198 (23.3%) responded to treatment by week 5; 501 (58.9%) did not respond and were rerandomized. The remaining 151 (17.8%) were not evaluated for R2 allocation, most often due to insufficient engagement (n = 112). Treatment disengagement by week 5 was significantly more likely for VBP (91 of 427 [21.3%]) than MBP (56 of 423 [13.2%]; Cramér *V* = 0.10; 95% CI, 0.03-0.13; *P* = .003).

Among those evaluated for R2 allocation (n = 699), there were no significant differences by week 5 between MBP and VBP on rates of response (MBP, 105 of 363 [28.9%]; VBP, 93 of 336 [27.7%]; Cramér *V* = 0.01; 95% CI, −0.06 to 0.08; *P* = .78) or remission (MBP, 76 of 361 [21.1%]; VBP, 54 of 327 [16.5%]; Cramér *V* = 0.05; 95% CI, −0.02 to 0.11; *P* = .16). Among those who also completed the PHQ-9 at week 12 (303 received MBP; 284 received VBP), there were no significant differences between MBP and VBP on rates of response (MBP, 144 of 303 [47.5%]; VBP, 134 of 284 [47.2%]; Cramér *V* < .001; 95% CI, −0.08 to 0.09; *P* = .99) or remission (MBP, 95 of 303 [31.4%]; VBP, 86 of 284 [30.3%]; Cramér *V* = 0.01; 95% CI, −0.07 to 0.09; *P* = .85).

The second condition (R2) was not significantly associated with response rates at week 12 among nonresponders (*P* = .43; Cramér *V* = 0.08). Response rates were 31.3% (36 of 115) for MBP nonresponders with weekly VBP vs 34.0% (33 of 97) for MBP nonresponders with monthly VBP (Cramér *V* = 0.08; 95% CI, −0.15 to 0.10; *P* = .43) and 26.0% (25 of 96) for VBP nonresponders with weekly VBP vs 36.4% (39 of 107) for VBP nonresponders with monthly VBP (Cramér *V* = 0.08; 95% CI, −0.02 to 0.23; *P* = .43). Furthermore, R2 was not significantly associated with remission at week 12: MBP plus weekly VBP (20 of 115 [17.4%]) and MBP plus monthly VBP (22 of 97 [22.7%]; Cramér *V* = 0.14; 95% CI, −0.16 to 0.06; *P* = .04; critical false discovery rate–corrected *P* = .03); weekly VBP plus MBP (9 of 96 [9.4%]) and monthly VBP plus MBP (25 of 107 [23.4%]; Cramér *V* = 0.14; 95% CI, 0.04-0.24; *P* = .04; critical false discovery rate–corrected *P* = .03).

### Depression Symptom Severity

Participants had moderate to moderately severe depression at baseline (mean [SD] PHQ-9 score, 15.0 [4.8]). When estimating PHQ-9 scores by R1 from baseline to week 5, scores improved with each successive week for VBP (*b* [SE] = −0.95 [0.05]; *P* < .001) (Table 2). There were no significant differences in slopes (*b* [SE] = −0.09 [0.07]; *P* = .23) or week 5 PHQ-9 scores (*P* = .29; *d* = 0.09).

**Table 2.** Parameter Estimates of Multilevel Models Estimating Change in PHQ-9 Scores From Baseline to Week 5 and From Week 6 to Week 12

Parameter	*b* (SE)	*P* value	Mean-estimated (SE) value	
			First time point	Second time point
**Mean (SE) values at baseline and week 5, respectively**				
PHQ-9 estimated by R1 from baseline to week 5				
Covariates				
Intercept (VBP)	14.37 (0.25)	<.001	14.37 (0.25)	9.65 (0.31)
Time	−0.95 (0.05)	<.001	NA	NA
MBP	0.03 (0.31)	.93	14.40 (0.25)	9.23 (0.30)
MBP × time	−0.09 (0.07)	.23	NA	NA
**Mean (SE) values at week 6 and week 12, respectively**				
PHQ-9 estimated by R2 from week 6 to week 12				
Covariates				
Intercept (VBP)	5.96 (0.51)	<.001	5.96 (0.51)	4.37 (0.58)
Time^a^	−0.26 (0.07)	<.001	NA	NA
MBP	−0.61 (0.70)	.38	5.34 (0.48)	4.29 (0.54)
MBP + weekly VBP	5.59 (0.65)	<.001	11.55 (0.42)	9.84 (0.48)
MBP + monthly VBP	4.65 (0.68)	<.001	10.60 (0.46)	8.79 (0.53)
Weekly VBP + MBP	5.70 (0.68)	<.001	11.65 (0.46)	10.70 (0.52)
Monthly VBP + MBP	5.48 (0.67)	<.001	11.43 (0.45)	9.43 (0.51)
MBP × time	0.09 (0.10)	.38	NA	NA
MBP + weekly VBP × time	−0.02 (0.09)	.83	NA	NA
MBP + monthly VBP × time	−0.04 (0.10)	.70	NA	NA
Weekly VBP + MBP × time	0.11 (0.10)	.28	NA	NA
Monthly VBP + MBP × time	−0.07 (0.10)	.47	NA	NA

Abbreviations: MBP, message-based psychotherapy; MBP + monthly VBP, rerandomized from MBP to MBP + monthly VBP; MBP + weekly VBP, rerandomized from MBP to MBP + weekly VBP; monthly VBP + MBP, rerandomized from weekly VBP to MBP + monthly VBP; NA, not applicable; PHQ-9, 9-Item Patient Health Questionnaire; R1, first randomization condition; R2, second randomization condition; VBP, video-based psychotherapy; weekly VBP + MBP, rerandomized from weekly VBP to MBP + weekly VBP.

^a^ Time centered at week 6.

**Figure 2.**
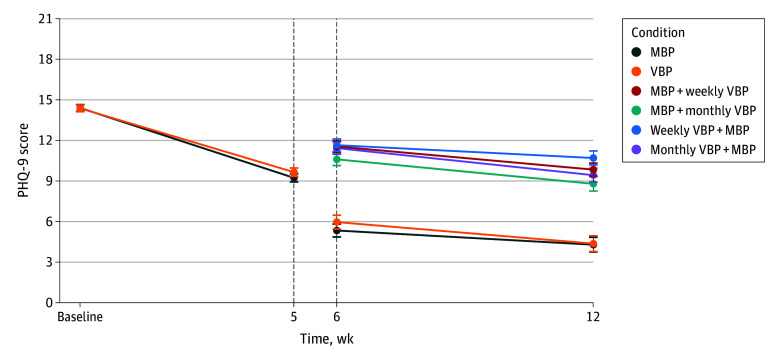
Nine-Item Patient Health Questionnaire (PHQ-9) Model-Estimated Means From Baseline to Week 5 by First Condition and From Week 6 to Week 12 by Second Condition MBP indicates message-based psychotherapy; and VBP, video-based psychotherapy. MBP: baseline to week 5, n = 423; weeks 6 to 12, n = 99. MBP + monthly VBP: participants rerandomized from MBP to MBP + monthly VBP (n = 108). MBP + weekly VBP: participants rerandomized from MBP to MBP + weekly VBP (n = 133). VBP: baseline to week 5, n = 427; weeks 6-12: n = 88. Monthly VBP + MBP: participants rerandomized from weekly VBP to MBP + monthly VBP (n = 115). Weekly VBP + MBP: participants rerandomized from weekly VBP to MBP + weekly VBP (n = 107). Error bars indicate SEs.

Baseline PHQ-9 scores were not significantly different between responders (mean [SD], 15.2 [4.8]) and nonresponders (mean [SD], 14.9 [4.8]; 2-sided *t* test *P* = .51). PHQ-9 scores were not significantly different between R2 groups from weeks 6 to 12: responders at weeks 6 (*b* [SE] = −0.61 [0.70]; *P* = .38; *d* = 0.15) and 12 (*P* = .92; *d* = 0.02); MBP nonresponders at weeks 6 (*d* = 0.19) and 12 (*d* = 0.21); VBP nonresponders at weeks 6 (*d* = 0.04) and 12 (*d* = 0.25) (Table 2). Slopes did not significantly differ between conditions (Table 2; Figure 2).

### Change in PHQ-9 Scores Moderated by Baseline Anxiety

At baseline, GAD-7 and PHQ-9 scores were moderately positively correlated (*r* = 0.57). In our moderation analysis, there was a significant main effect of time (*b* [SE] = −0.73 [0.04]; *P* < .001), and higher baseline anxiety was associated with greater improvement on depression from baseline to week 12 (*b* [SE] = −0.01 [0.01]; *P* = .006) (Table 3). The main effect of MBP, 2-way interactions between time × MBP and GAD-7 × MBP, and the 3-way interaction between time × GAD-7 × MBP were not significant (Table 3).

**Table 3.** Parameter Estimates of Multilevel Model Estimating Change in PHQ-9 Scores From Baseline to Week 12 Moderated by Mean-Centered Baseline GAD-7 Score

Covariate	*b* (SE)	*P* value
Intercept (VBP)	14.12 (0.21)	<.001
Time	−0.73 (0.04)	<.001
Spline	0.58 (0.06)	<.001
MBP	−0.04 (0.26)	.89
Baseline GAD-7score^a^	0.47 (0.04)	<.001
Time × MBP	−0.10 (0.06)	.09
Spline × MBP	0.11 (0.09)	.20
Baseline GAD-7 score × MBP	−0.004 (0.05)	.93
Time × baseline GAD-7 score	−0.01 (0.01)	.006
Time × baseline GAD-7 score × MBP	0.002 (0.01)	.78

Abbreviations: GAD-7, 7-Item Generalized Anxiety Disorder Scale; MBP, message-based psychotherapy; PHQ-9, 9-item Patient Health Questionnaire; VBP, video-based psychotherapy.

^a^ Mean-centered (mean [SD], 12.12 [5.12]).

### Social Functioning

Social functioning improved for both MBP and VBP from baseline to week 12 (eTable 1 in Supplement 2). Video-based psychotherapy participants significantly improved by 0.34 points with each week (*b* [SE] = 0.34 [0.04]; *P* < .001). There were no significant differences in slopes (*b* [SE] = 0.05 [0.06]; *P* = .42) or scores at weeks 5 (*d* = 0.001) or 12 (*d* = 0.13).

### Therapeutic Alliance

The multilevel model examining change in therapeutic alliance ratings from weeks 4 to 10 by R2 condition (eTable 2 and eFigure in Supplement 2) indicated that there were no significant differences at weeks 4 or 10 between MBP and VBP responders (week 4, *d* = 0.18; week 10, *d* = 0.23), MBP nonresponders who were rerandomized to also receive weekly or monthly VBP (week 4, *d* = 0.08; week 10, *d* = 0.02), or VBP nonresponders who were rerandomized to also receive MBP with weekly or monthly VBP (week 4, *d* = 0.02; week 10, *d* = 0.05). However, at week 4, VBP nonresponders reported significantly lower therapeutic alliance than MBP responders (weekly VBP plus MBP vs MBP: *d* = 0.41; *P* = .008; monthly VBP plus MBP vs MPB: *d* = 0.38; *P* = .005) or VBP responders (weekly VBP plus MBP vs VBP: *d* = 0.56; *P* < .001; monthly VBP plus MBP vs VPB: *d* = 0.53; *P* < .001), and MBP nonresponders had significantly lower therapeutic alliance than MBP responders (MBP plus weekly VBP vs MBP: *d* = 0.92; *P* < .001; MBP plus monthly VBP vs MPB: *d* = 0.94; *P* < .001) or VBP responders (MBP plus weekly VBP vs VBP: *d* = 1.03; *P* < .001; MBP plus monthly VBP vs VPB: *d* = 1.06; *P* < .001) and VBP nonresponders (MBP plus weekly VBP vs weekly VBP plus MBP: *d* = 0.57; *P* < .001; MBP plus weekly VBP vs monthly VBP plus MPB: *d* = 0.52; *P* < .001; MBP plus monthly VBP vs weekly VBP plus MBP: *d* = 0.54; *P* < .001; MBP plus monthly VBP vs monthly VBP plus MBP: *d* = 0.48; *P* < .001).

Although therapeutic alliance ratings increased across all conditions by week 10, these changes were not statistically significant. At week 10, both VBP nonresponders and MBP nonresponders continued to report significantly lower therapeutic alliance than responders (weekly VBP plus MBP vs MBP: *d* = 0.53; *P* < .001; monthly VBP plus MBP vs MBP: *d* = 0.44; *P* = .002; weekly VBP plus MBP vs VBP: *d* = 0.73; *P* < .001; monthly VBP plus MBP vs VBP: *d* = 0.62; *P* < .001; MBP plus weekly VBP vs MBP: *d* = 0.70; *P* < .001; MBP plus monthly VBP vs MBP: *d* = 0.76; *P* < .001; MBP plus weekly VBP vs VBP: *d* = 0.86; *P* < .001; MBP plus monthly VBP vs VBP: *d* = 0.93; *P* < .001). Video-based psychotherapy nonresponders reported higher therapeutic alliance than MBP nonresponders at week 10, with significantly higher ratings for monthly VBP plus MBP compared with MBP nonresponders (monthly VBP plus MBP vs MBP plus weekly VBP: *d* = 0.28; *P* = .01; monthly VBP plus MBP vs MBP plus monthly VBP: *d* = 0.28; *P* = .03), and for weekly VBP plus MBP than MBP plus weekly VBP (*d* = 0.25; *P* = .04). There were no significant differences in therapeutic alliance ratings at week 10 between MBP plus monthly VBP and weekly VBP plus MBP (*d* = 0.25; *P* = .07).

### Quality of Care and Amount Helped

There were no significant differences in ratings on quality of care or amount helped between R2 responders (*d* = 0.31 and *d* = 0.26, respectively), MBP nonresponders (*d* = 0.12 and *d* = 0.12, respectively), or VBP nonresponders (*d* = 0.08 and *d* = 0.14, respectively) (eTable 3 in Supplement 2).

### Goal Satisfaction, Continued Use, and Recommendation to Others

There were no significant differences in week 12 ratings on goal satisfaction or plans to continue using treatment between R2 groups (eTables 4 and 5 in Supplement 2). However, VBP participants were significantly more likely to recommend treatment at week 12 compared with MBP participants (VBP, 69 of 71 [97.2%]; MBP, 70 of 80 [87.5%]; odds ratio, 0.18; 95% CI, 0.04-0.88; *P* = .03) (eTable 6 in Supplement 2). Message-based psychotherapy nonresponders with weekly VBP (74 of 106 [69.8%]) were significantly more likely to recommend treatment than those with monthly VBP (54 of 92 [58.7%]; *P* = .04); there was no significant difference between VBP nonresponders receiving weekly vs monthly VBP.

Post hoc analyses indicated that response to treatment was significantly associated with satisfaction ratings at week 4 (Cramér *V* = 0.18-0.29; *P* < .001). Higher proportions of responders than nonresponders reported goal satisfaction (responders, 139 of 177 [78.5%]; nonresponders, 201 of 437 [46.0%]), plans to continue using treatment (responders, 169 of 180 [93.9%]; nonresponders, 340 of 435 [78.2%]), and recommending treatment (responders, 166 of 180 [92.2%]; nonresponders, 325 of 437 [74.4%]) at week 4.

## Discussion

To our knowledge, this is the first study comparing MBP with VBP and their combination in a pragmatic sequential multiple assignment randomized clinical trial of patients with depression seeking care from a commercial platform. We hypothesized that MBP would be superior due to immediacy of care delivery, facilitation of increased therapeutic contact, and creation of records to facilitate action plan implementation^15,16,17^; however, our data did not support this hypothesis. Nonetheless, the results support the use of MBP, given similar outcomes between MBP and VBP and significant improvements in depression symptoms and social functioning. For nonresponders, combinations of MBP and VBP were not associated with improvement beyond either modality alone. The data suggest that MBP is a viable depression treatment, but do not support using a combination of interventions to improve service delivery for those who do not show early response.

Although MBP has the potential to transform therapy, its reach is often constrained by insurance policies. Coverage is a decisive factor in treatment choices. Before the COVID-19 pandemic, when few insurers reimbursed for video-based care and most of the clients of this study’s digital mental health platform paid out of pocket, more than 75% chose MBP over VBP. As the digital mental health platform shifted to a predominantly insurance-based model in response to the pandemic, VBP became the primary mode of care. Expanding insurance coverage will be essential to ensure widespread access.

### Effect of Anxiety on Outcomes

Data did not support our hypothesis that patients with comorbid anxiety would respond best to MBP. We did find that higher baseline anxiety was associated with greater improvement in depression, which is inconsistent with other studies.^49,50^ Because baseline anxiety and depression were moderately correlated, perhaps those with higher baseline anxiety had higher depression and therefore had more statistical room for improvement. This analysis raises a question about whether other diagnoses may moderate the effectiveness of MBP or VBP. For instance, the immediacy of treatment that characterizes MBP may be contraindicated when treatment requires strict boundary setting. Diagnosis as a moderator of MBP is an area for future study.

### Treatment Engagement

Most consumers engage with digital mental health platforms for less than 2 months.^51^ In an earlier observational study conducted with data from the digital mental health platform, more than 50% of patients improved to below the clinical cutoff by week 6, but 37% of clients had disengaged from treatment by this time point.^51^ In the present study, treatment disengagement by week 5 was 8 percentage points higher for participants receiving VBP than MBP, a modest but statistically significant difference, even though VBP participants had similar outcomes. The structure of VBP may be a barrier to ongoing engagement; people seeking psychotherapy can find weekly scheduled meetings inflexible.^52,53,54,55,56,57,58,59,60,61,62,63,64^ Participants who have improved or feel they are not benefiting may be particularly likely to discontinue, perceiving these barriers to outweigh the potential for further benefit. We hypothesized that MBP would result in better outcomes because the structure of MBP may better align with participants’ needs and simplify therapy logistics. Although we did not find differences in outcomes, this modest difference in engagement is consistent with our hypotheses.

Therapeutic alliance ratings did not significantly improve from week 4 until week 10 for any group. Nonetheless, week 10 therapeutic alliance ratings for those originally receiving MBP who were reassigned to supplemental VBP approached ratings of participants originally receiving VBP, supporting the premise that video-based care was associated with greater alliance. However, there were no differences between those who received monthly or weekly VBP. Dosage at these levels may not have as strong of an effect on alliance as simply receiving any VBP. Future research might explore the value of intentionally engaging in alliance-building efforts early in treatment by incorporating a video session, articulating shared goals, offering choices on therapeutic approach, strengthening emotional bonds, and regularly inviting feedback.

### Limitations

This study has some limitations. Several studies have shown that VBP is a robust active comparator with similar outcomes and better engagement than in-person therapy.^12,13,14^ For this reason and due to ethical concerns, we did not use a waiting list, no-treatment control. Given that participants improved with no differences between treatments, we cannot exclude the possibility of regression to the mean. However, outcomes were greater than usual care conditions of clinical trials for depression, where spontaneous remission of untreated depression is 23% over 3 months,^4^ compared with about 31% in our study. Missing data may have affected the robustness of some findings.

## Conclusions

In this sequential multiple assignment randomized clinical trial comparing MBP with VBP, there were no differences between groups on improvement in depression or social functioning. More participants in the VBP group disengaged by week 5, and there were some indicators that VBP led to greater therapeutic alliance. There were no differential effects on depression after rerandomizing nonresponders. These findings were consistent with prior studies that MBP is a viable alternative to video or face-to-face treatment. Currently, however, most insurance companies do not reimburse for message-based care, limiting the widespread implementation of this likely preferred and effective approach.

## Notes

Supplement 1.
Trial Protocol and Statistical Analysis Plan

Supplement 2. **eMethods 1.** Identification and Management of Suspected Fraudulent Participation **eMethods 2.** Data Management Statement **eMethods 3.** Intention-to-Treat Procedures **eMethods 4.** Supplemental Measure Descriptions **eMethods 5.** Coding of Therapist Value **eTable 1.** Parameter Estimates of Multilevel Model Predicting Change in Neuro-QOL Scores From Baseline to Week 12 by First Condition **eTable 2.** Parameter Estimates of Multilevel Model Predicting Change in WAI-SR Scores From Week 4 to Week 10 by Second Condition **eFigure.** WAI-SR Model-Estimated Means From Week 4 to Week 10 by Second Condition **eTable 3.** Parameter Estimates of Multilevel Models Predicting Ratings on Quality of Care and Amount Helped at Week 10 by Second Condition **eTable 4.** Parameter Estimates of Multilevel Mixed Effects Logistic Regression Model Predicting Goal Satisfaction at Week 12 by Second Condition **eTable 5.** Parameter Estimates of Multilevel Mixed Effects Logistic Regression Model Predicting Plans to Continue Using Treatment at Week 12 by Second Condition **eTable 6.** Parameter Estimates of Multilevel Mixed Effects Logistic Regression Model Predicting Recommendation of Treatment to Others at Week 12 by Second Condition
eReferences.

Supplement 3.
Data Sharing Statement
