# Supplementary material for: Message-Based vs Video-Based Psychotherapy for Depression: A Randomized Clinical Trial
Source: JAMA Netw Open. 2025 Oct 30;8(10):e2540065. doi: 10.1001/jamanetworkopen.2025.40065 (PMC12576495; doi:10.1001/jamanetworkopen.2025.40065)
Supplement: Supplement 1. — Trial Protocol and Statistical Analysis Plan [file jamanetwopen-e2540065-s001.pdf]

Assessing the Dynamic Application of Psychotherapy by Texting (ADAPT)
Trial Protocol and Statistical Analysis Plan

Table of Contents

Section No.	Title	Page No.
1.1	Background and Significance	1
1.2	Study Aims	2
1.3	Study Description	2
2.0	University of Washington Study Team	2
3.0	Study Design	3
4.1	Setting	3
4.2	Participants	3
4.3	Recruiting and Screening	4
4.4	Inclusion and Exclusion Criteria	5
4.5	Costs and Payment to Participants	6
4.6	Study Enrollment Procedures	6
5.1	Procedures	7
5.2	Study Assessments	8
6.0	Safety and Data Management	9
7.0	Secondary Use or Sharing of Information or Contact Information	11
8.0	Statistical Analysis Plan	11

1.1 Background and Significance

The delivery of psychotherapy as it is currently designed is inaccessible to most patients in need of care and is not provided as frequently as recommended, reducing its clinical impact. Advances in technology and ubiquitous ownership of mobile technology can be leveraged as novel methods of delivery to move the needle on rates of depression in the US.

Teletherapy is the use of mobile and communication technology to provide psychotherapy services at a distance. This is a method of psychotherapy delivery that has been in existence for nearly 40 years, originally as telephone-based care (1980's), then as videoconferencing-based care (2000's), and more recently as self-guided smartphone applications and internet programs and message-based (text and video messages) care. Research has consistently shown videoconferencing-based psychotherapy (VBP) to be an engaging form of delivery that enhances access equality, has high levels of patient satisfaction and is comparable to conventional treatment among diverse patient populations, ages and diagnostic groups. It is considered to be equivalent to and more accessible than traditionally delivered psychotherapy and is currently reimbursed under telehealth parity laws in as many as 36 states. However, many clinicians still harbor negative views of VBP, making access to this care limited. It is also delivered in the same way as in-person psychotherapy, with once-weekly meetings generally scheduled during the work week. Although transportation needs are mitigated, time demands are not. Thus, VBP, while effective, is challenged by a limited workforce and a limited therapeutic structure that is less engaging.

Message-based psychotherapy (MBP), defined as the use of text messages or asynchronous voice/video messaging, is an interesting alternative to VBP and self-guided psychotherapy (SGP). It differs from VBP in that patient-therapist contact can be done asynchronously, as often as needed, and like SGP, can be delivered when it matters most to the patient. However, like VBP, care is guided by licensed therapists who can intervene quickly when a patient is struggling with a problem or needs more support and guidance with a therapeutic strategy. Studies show that asynchronous messaging between therapists and patients support action plan implementation, results in better therapeutic alliance and overall adherence to treatment recommendations by patients, and in small studies, results in significant reductions in depression. MBP is based on the theory that interventions have their biggest clinical impact when the consumer is at their greatest level of receptivity, and through better timing and tailoring, improve the consumers' readiness to change, self-efficacy to implement the intervention, and goal-directed behavior. MBP is becoming a widely available form of psychotherapy delivery, with as many as 20 companies providing MBP. MBP, like VBP, is reimbursable under some health plans, and small sample proof of concept studies found MBP to be engaging and effective, both alone and in combination with VBP.

MBP has yet to be rigorously evaluated for its effectiveness overall, and there is no guidance as to who should receive MBP alone or in combination with VBP. There is no research that has addressed when MBP should be offered alone or in conjunction with VBP, and who would respond (or not respond) to either alone or in combination. In sum, MBP may be the next, impactful, and novel method of psychotherapy delivery, but is in further need of study.

We propose that MBP may be a more potent intervention than weekly VBP alone because of (1) the frequency of contact between patients and therapists, (2) the relevance of therapeutic intervention at the time of its delivery, and as a result, (3) improved working alliance by patients.

This study will have commercial impact in that it will be the first to determine the relative effectiveness of unlimited, asynchronous MBP compared to more traditional VBP. Thus, this study will make a significant public health impact by being the first to evaluate a commercial product delivering MBP compared to VBP and addresses NIMH strategic priority area 4.1 (improve the efficiency and effectiveness of mental health services through research). Second, this study will have an impact on clinical care in that it will be able to determine the best digital treatment algorithm for people who do not respond to either MBP or VBP only and who respond to VBP, MBP, or the combination. There has been considerable interest in the clinical and policy realm in understanding who responds to different levels of care, and when patients should be "stepped up" in intervention intensity. Thus, this study will make a significant clinical impact by identifying a treatment sequence for systems that provide teletherapy and is responsive to NIMH priority area 3.2 (develop ways to tailor existing and new interventions to optimize outcomes).

1.2 Study Aims
This fast-track Small Business Innovations Research (SIBR) aims to (1) determine the relative effectiveness of unlimited MBP compared to weekly VBP, (2) determine whether a switch to monthly VBP+MBP or weekly VBP+MBP is better for people who do not respond to VBP or MBP alone, (3) determine who responds to best to VBP, MBP, or the combined conditions, and (4) test the assumptions about the frequency and timeliness of treatment on working alliance and hence treatment outcomes.

1.3 Study Description
In Phase 2, we will conduct a large-scale clinical trial using a 12-week longitudinal sequential multiple assignment randomized trial (SMART)[1] design to test the aims outlined in Section 1.2. We will aim to test the relative effectiveness of psychotherapy delivered via messaging-based platforms compared to video-based care, as well as determine the best method of enhancing care for those who do not respond to either daily messaging only or traditional, once-a-week video-based sessions.

2.0 University of Washington Study Team
All members of the University of Washington (UW) study team have completed CITI and HIPAA trainings. The study team meets multiple times per month to review study procedures, progress, and implementation.

<u>Principal Investigator</u>:
Study Roles: Oversee study procedures, primary responsibility for regulatory tasks and recruitment.
Experience: Professor in Psychiatry and Behavioral Sciences and a licensed clinical psychologist. The PI is also a member of the NIMH Advisory Council. For the past 20 years, the PI has been actively researching the efficacy, effectiveness, and implementation of behavioral interventions for depression, specifically focused on Behavioral Activation and Problem-Solving Treatment. The PI has recently added to research on the development of targeted interventions that have the greatest potential for being implemented after efficacy and effectiveness are determined. The PI's interest in access to high-quality care and streamlining treatment led her to consider the potential for neuroscience and technology to rapidly innovate and streamline how we treat mental illness.

<u>Program Manager</u>:
Study Roles: Oversee study procedures, ensure timely implementation of study procedures by Talkspace staff, responsibility for all regulatory tasks.
Experience: Licensed clinical social worker with experience delivering and training on structured interventions; member of CREATIV Lab for 3+ years; previous and ongoing experience in clinical trial management.

Research Coordinator:
Study Roles: Develop and disseminate recruitment ads, assist the Program Manager in the implementation of study procedures, and provide technical assistance to Talkspace staff as needed.
Experience: Member of CREATIV Lab for 5 years; previous and ongoing experience with research recruitment, study implementation, and clinical trials.

Research Study Coordinators:
Study Roles: Primary responsibility for tracking assessment completion and will also assist Program Manager and Research Coordinator in recruitment and implementation of study procedures.
Experience: Member of CREATIV Lab for 2 years. RSC fulfills this role in another pilot study with Talkspace and other clinical trials.

Study Therapists:
Study Roles: Deliver therapy on Talkspace's platform to study participants.
Experience: Licensed mental health clinicians.

3.0 Study Design
We will use a sequential multiple assignment randomized trial (SMART) design.

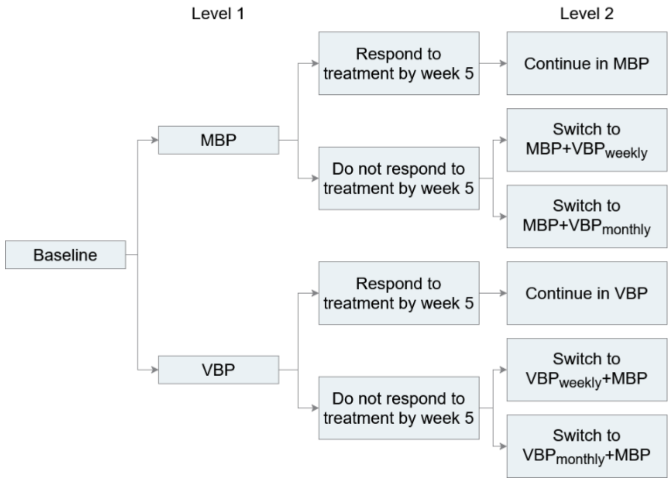

LEVEL 1 Randomization: Participants will be randomized to either MBP or weekly VBP. After six weeks of treatment, participants will move to Level 2.

LEVEL 2 Randomization: Those who have made a 50% reduction to Patient Health Questionnaire-9 (PHQ-9)[2] scores to their assigned treatment will continue in that same treatment for 6 more weeks. Participants randomized to VBP who do not show 50% reduction in PHQ-9 scores will be randomized to either weekly VBP augmented with unlimited MBP (augmentation arm) or monthly VBP with unlimited MBP (switch arm). Participants randomized to MBP who do not show a 50% reduction in PHQ-9 scores will be randomized to either unlimited MBP with weekly VBP (augmentation arm) or unlimited MBP and monthly VBP (minimal augmentation). The decision to switch or augment treatment after there is no response to treatment (<50% change in PHQ-9 score) is based on treatment recommendations from the American Psychological Association, The National Health Service in the UK, and the AHRQ depression guidelines. Talkspace therapists, operating as consulting members of the study team, will complete the Clinical Global Impression – Improvement Scale[3] after 5 weeks of treatment; if the participant does not complete a PHQ-9 in any one of the weekly assessments at weeks 3, 4, or 5 in the study timeline, this scale will be referenced for determining appropriateness for Level 2 randomization.

4.1 Setting
This study is fully remote as the study is concerned with evaluating text and video-based psychotherapy, an innovative care delivery platform that is becoming widely available but has not been adequately evaluated in a clinical trial.

Talkspace provides MBP and VBP via a proprietary, HIPAA-compliant mobile application via text, audio, and video messages. Talkspace is the ideal platform to study the effectiveness of MBP compared to VBP and to identify methods to improve care for those who do not respond to MBP or VBP. Talkspace provides services throughout the US; they match therapists and clients from the same state.

4.2 Participants
Estimated sample size of 1,000 participants.

4.3 Recruiting and Screening

Recruitment Sources: We will be recruiting from four primary sources: (1) traditional advertisements using newspaper, radio and podcast ads and stories, (2) social media sources such as Craigslist, Facebook, Twitter, and Instagram, (3) recruitment from people who are seeking care from Talkspace, and (4) advertisements on mental health sites such as Mental Health America. Potential participants will complete a screener survey hosted on REDCap.[4,5] The study will also recruit from Prolific (starting late 2022) and ResearchMatch (starting early 2023).

Prolific Recruitment: For the Prolific recruitment survey, we will be using Prolific, an online recruitment tool for research. Users log in and complete research surveys and are paid an average of $8.00 per hour for completing surveys. The survey is confidential. Prolific users will self-identify by responding to the task. After a participant self-identifies as wanting to complete the task, they will be directed to an online REDCap survey. They will first review an information statement that contains all necessary elements of consent. After reviewing that text, if they decide to continue, they will complete a brief survey. Survey respondents who answer either "More than half the days" or "Nearly every day" to two or more of questions 5-8 will see information about the broader ADAPT study, including a link to the study. The information that will be shown is the same information included in recruitment materials previously approved by the IRB. We expect that responding to the survey will take up to 1 minute. After completion of the measures, each participant will be routed back to Prolific to certify completion of the survey. Payment for survey completion is handled by Prolific. If survey respondents choose to enroll in the ADAPT study, there will be no ability to link responses to the recruitment survey to any information gathered in the ADAPT study. All communication with survey respondents will take place within the Prolific platform.

ResearchMatch Recruitment: ResearchMatch is a national electronic, web-based recruitment tool that was created through the Clinical & Translational Science Awards Consortium in 2009. A Principal Investigator (PI) or a PI-approved recruitment proxy will request recruitment access to ResearchMatch by providing IRB approved protocol and recruitment material. The Institutional Liaison will set access to ResearchMatch to the expiration date on the IRB protocol. Either the PI or the PI-approved recruitment proxy will query the ResearchMatch database to find volunteers that match their study criteria. Researchers will send out IRB-approved content to potential matches through ResearchMatch. The study's recruitment content is inserted into the standard ResearchMatch electronic notification which informs matched volunteers that a researcher has identified them as a potential match for their study. The study recruitment message will not include the study's direct contact information (e.g., email, phone). Potential matches receive recruitment content and then opt in to having ResearchMatch share their contact information with the study team. Contact information for ResearchMatch volunteers will only be shared with the study team about potential volunteers who have agreed to such a release. Only after a volunteer responds yes to the recruitment message will ResearchMatch release their contact information to the researchers who then follow the standard enrollment and informed consent process. Once the study team receives contact information for interested participants, a member of the study team will email the participant with information on the study and how to get started.

Recruitment Materials: Flyers will be used to recruit participants from traditional advertisement sources such as newspapers, radio, and magazines; and social media sources such as Craigslist, Facebook, Twitter, LinkedIn, and Instagram. We will also collaborate with our partners from other projects to publicize the study, including Mental Health America. A consent script that includes an introduction to the project will be used to recruit participants seeking care from Talkspace. For the Prolific recruitment survey, the survey will be posted as a task on Prolific. We will distribute a recruitment message to the ResearchMatch network.

Eligibility screening: Regardless of where the potential participant learns about the study, all potential participants will be screened for the study by completing a survey hosted by REDCap that will connect the potential participant to a consulting therapist (a Talkspace staff member who completes intake for all Talkspace services) in Talkspace's HIPAA compliant, encrypted text platform. All patients seeking services from Talkspace complete a PHQ-9 during their initial screening. If a patient meets eligibility criteria for the study, the consulting therapist will ask if they wish to learn more about the study. If so, the consulting therapist proceeds with the consent procedures. If the potential participant does not meet criteria for the study, the consulting therapist will discuss alternate treatment options with the potential participant. The PHQ-9 scores collected on Talkspace will not be retained as part of study data. Participants will provide information needed to assess eligibility including date of birth, location, PHQ-9, phone number, email address, and primary language prior to enrollment. This information is retained as part of study data.

4.4 Inclusion and Exclusion Criteria

Inclusion criteria: 18 years old and older, English and/or Spanish speaking, living in the US in a state where a participating licensed Talkspace clinician is available, PHQ-9 score ≥10.

Prolific recruitment survey: Prolific users who are 18 years old and older, English speaking, living in the US in a state where a participating licensed Talkspace clinician is available.

Unavailable therapist: Potential participants who are eligible at an initial baseline but unable to enroll in the study due to lack of therapist availability are contacted by Talkspace via email to let them know. In that email, they are offered immediate care at Talkspace and are told that they will be contacted when a study therapist is available, unless they opt out of further communication. Once a study therapist is available in their state, any waiting potential participants are either randomized or, if it has been more than 30 days since their initial eligibility baseline, they are sent a link to complete a new baseline. All eligibility criteria should still be met at the new baseline in order for the participant to be randomized. However, participants with PHQ-9 scores between 5 and 10 may be randomized if they remain interested in participating in the study. No one with a PHQ-9 score <5 will be randomized. The study team removes recruitment materials from states with no therapist capacity, so these situations should be limited to individuals that enter the enrollment pipeline while space is available but do not complete the enrollment activities prior to a therapist's caseload filling.

Exclusion criteria: Under 18 years of age, does not speak English or Spanish, PHQ-9 <10, and/or lives in a state without an available Talkspace clinician. Individuals with active suicidal ideation, homicidal ideation, or risk of harm to others will be excluded and referred to appropriate care. Individuals who self-identify as taking antipsychotic or mood stabilizer medications, or who have been prescribed Lithium medication will be excluded. Individuals who identify as having been prescribed medication for having "a period of being so excited or irritable that you got into trouble, or your family or friends worried about it" will be excluded. Individuals who indicate that a doctor has said they were manic-depressive or had bipolar disorder, or that they have schizophrenia, a schizoaffective disorder or psychosis will be excluded. Individuals who indicate that they would have a hard time using either video or message-based care will be excluded. Individuals who indicate that they would not be willing to complete daily and weekly surveys in addition to Talkspace treatment will be excluded. Prior to early 2023, all recruitment to date for the study took place via advertisements on mental health platforms like Mental Health America. In early 2023, the study will expand recruitment to social media and other recruitment avenues. With this expansion, the study team would like to be proactive in implementing procedures that will protect again fraudulent participants, including the following:

- Exclusion of potential participants with a mismatch between age and date of birth: any potential participant whose provided age and provided date of birth do not match will be excluded from the study. They will receive an email stating that they are ineligible for the study; included in the email will be a statement that they can contact the study team if they believe the ineligibility determination is in error.

- Other potential indicators of fraudulent participation include fake names (e.g., random letters, inanimate objects), fake email addresses, patterns of enrollment behavior (e.g., numerous registrations from same state in short period of time, same information submitted across numerous records), and nonsensical qualitative responses. We request flexibility and researcher discretion to evaluate records of potential fraudulent participation and exclude those participants whose records contain signs of fraudulent activity. Our lab has extensive experience in the assessment and determination of fraudulent research participation, including STUDY00004997 and STUDY00012455, and base these determinations on both those experiences and an understanding of the literature in this area.[6-9] Any potential participant excluded will receive an email stating that they are ineligible for the study; included in the email will be a statement that they can contact the study team if they believe the ineligibility determination is in error.

- Participants may also be identified to meet fraudulent criteria after starting treatment in the study. This may occur when communicating via email with the study team by providing inconsistent personal information (e.g., birth date) to the information they submitted when screening for the study, or if inconsistent data are found across multiple screeners that have been submitted by the same individual.

4.5 Costs and Payment to Participants

Costs to Participants: Participants will not pay the Talkspace service fee, nor will they pay for care during their time in the trial. Participants will be alerted in the consent that using their mobile device for treatment and/or questionnaires may count against their data plan and are encouraged to either complete while on Wi-Fi or using a computer to avoid if necessary.

Payments: Based on learnings from Phase 1, participants in Phase 2 will receive up to $125 for completing assessments during their 12 weeks in the study. Participants earn incentive payments for completion of the weekly assessments. Payments will be sent at 3 time points in the study: after the completion of week 4, week 8, and week 12. Participants who choose to withdraw from the study early and therefore will not be eligible to receive the full $125 will be given the option to complete a 1-question Early Exit Survey and receive $3 for the completion of this survey (Table 1).

Table 1. Study Compensation

1st payment: after end of week 4	Baseline Assessment	$7
	Week 1 Assessment	$7
	Week 2 Assessment	$7
	Week 3 Assessment	$7
	Week 4 Assessment	$7
2nd payment: after end of week 8	Week 5 Assessment	$10
	Week 6 Assessment	$10
	Week 7 Assessment	$10
	Week 8 Assessment	$10
3rd payment: after end of week 12	Week 9 Assessment	$12.50
	Week 10 Assessment	$12.50
	Week 11 Assessment	$12.50
	Week 12 Assessment	$12.50
	Early exit survey for dropouts	$3

Non-monetary Compensation: In Phase 2, participants will receive non-monetary compensation in the form of GIFs, facts, insights, days off from surveys, etc.

4.6 Study Enrollment Procedures

Consent Procedures: The informed consent form will be completed via REDCap, with questions directed to the study team as needed. Participants are encouraged to contact the study team via email if parts of the informed consent form are unclear, or if additional information is desired. Participants will have the ability to ask questions of the study staff during initial consent activities and will have the ability to send questions to the study team via phone and/or email. Participants will be told they will be randomized to one of two levels of care. They will be told to use the treatment they are assigned as indicated (either weekly VBP appointments or unlimited MBP) for 6 weeks, and that they will complete daily and weekly surveys about their mood and functioning for 6 weeks. Participants will be told that 6 weeks after care, they will either continue with their treatment as assigned if they are seen to be responding or will be randomized to receive additional care if they are not responding to care. Finally, users will be told that participation at any point in the study is completely voluntary. Participants must select “I consent” at the end of the consent form in order to move forward with study procedures. Individuals who agree to participate will be identified in Talkspace’s electronic health record as taking part in the study so that therapists can adhere to the study protocol.

Consent will also be obtained for recruitment via Prolific survey. Prior to answering any questions, participants will review an informational statement that details the purpose of the study, what participation entails, and other elements of consent. No consent will be obtained for recruitment that occurs outside of the Prolific platform or ResearchMatch.

Comprehension: Participants will be asked to complete a consent quiz to ensure they understand the study protocol. If a participant responds incorrectly to any item on the consent quiz, that section of the consent will be reviewed. Consent questions are all TRUE/FALSE format and are:

1. I get to choose the type of treatment I receive as a participant in this study.
2. I will be randomly assigned my treatment plan at the start of the study.
3. I will be completing questionnaires throughout the study and will receive Amazon gift codes for answering those questions.
4. I am a volunteer and can drop out of the study at any time, without risk of losing access to available care.

Electronic presentation of consent information: Participants will receive a copy of the consent form via email. The aim of this study is to learn about the acceptability and effectiveness of message-based care. Therefore, all participants must be willing to utilize technology to access the study treatment and assessments. If necessary,

additional information during the research (e.g., any significant new findings, including risk information) will be shared through SMS messaging, email and/or phone.

5.1 Procedures
During the baseline assessment, participants respond qualitatively to the following question: "How would you describe the top issue you would like help with in therapy? Describe the problem as something that is potentially changeable in therapy. So rather than writing 'Going through a divorce' you might write 'Feeling sadness and anger while I'm going through a divorce.'" Upon participant completion of this field, REDCap will send an automated alert to the UW study team that information has been entered in this field. A clinical member of the UW study team will review this information within 1 business day. If actionable information (e.g., risk of harm to self or others) is shared in that field, the UW study team will alert the Talkspace study team, and the participant will not be enrolled in the study. Because this information is provided prior to randomization and thus prior to assignment to a clinician, the Talkspace study team will connect the individual to a clinician to complete a full assessment and escalate through Talkspace's established crisis procedures.

<u>Identification and Management of Suspected Fraudulent Participation</u>: Consented participants who have not yet started treatment and are identified post-consent to meet fraudulent criteria are contacted via email notifying them of their removal from the study. In the consent form, participants are informed that they may contact the study team to receive a detailed explanation as to why their data did not reach study standards. Participants who have started treatment in the study and are later identified to meet fraudulent criteria are contacted via email notifying them of their removal of treatment and from the study. The participant's assigned therapist is also notified via email about the participant's removal from the study.

<u>Randomization</u>: See Section 3.0. Baseline Level 1 randomization to MBP or VBP occurred on REDCap and was triggered by a research coordinator upon enrollment. We used simple randomization with pre-specified and concealed blocks by therapist. Level 2 randomization occurred on REDCap. Level 1 participants who responded to treatment continued in their baseline condition. Level 1 participants who did not respond to treatment by week 5 (PHQ-9 score > 50% of baseline PHQ-9 score, or CGI-I score >2) were re-randomized to receive MBP and supplemental weekly or monthly VBP.

<u>Treatment Procedures</u>: All procedures described here are methods developed and used by Talkspace, unless otherwise specified. There are four interventions in this study: Level 1 interventions are unlimited MBP and weekly VBP. Level 2 interventions are MBP with monthly VBP, and MBP with weekly VBP. After participants give consent to participate, they will be randomized to one of the two Level 1 conditions. As per Talkspace's model, participants are offered a choice of three therapists, based on the state the therapist is licensed in. Procedures for consumer/clinician initiation are the same for each treatment option. Participants can contact their therapists any time after they are provided with choices. Once participants reach out to a therapist, therapists connect within 15 minutes to 24 hours, depending on the time of day the participants made their choice and the therapist's office hours. All therapists maintain office hours, typically 9-6 in their time zone, 5 days a week (typical to private practice models of care). Talkspace patients are offered an opportunity to participate in a complementary, but not compulsory, live video session with their chosen therapist. Therapists are required to offer it, but historically, only 24% of patients avail themselves of this service. Although patients can use Talkspace services for as long as they and the therapist feel it is necessary, on average, patients use 10 weeks of care, and for the purposes of this study, participants will be told they have 12 weeks of care available to them.

<u>Assessment Procedures</u>: Participants will continue to complete Talkspace's standard assessments (PHQ-9 and Generalized Anxiety Disorder-7 [GAD-7][10]), which are delivered every 3 weeks. For the 12 weeks that the participant is in the study, these assessments will be shared with the study team.

All assessments discussed in the following section will be completed using REDCap. Participants will receive links to complete the assessments via text message using Twilio, a REDCap extension that allows for SMS communication with participants. Participants will complete a baseline assessment, 2 follow-up assessments (6- and 12-weeks), daily ratings, and weekly assessments. Participants are given a 3-day window to complete a brief assessment of mood and improvement (weekly assessment) and a follow-up assessment of mood, functioning, cognition, improvement and satisfaction. If a participant does not complete the weekly or follow-up assessment within 24 hours, the participant will receive a reminder SMS message. If participants do not complete the

assessment within 24 hours of the reminder, an email reminder is sent. REDCap provides the project coordinator with daily information about research participants' completion of assessments. These data will be used to assess the utility of the study reminder system in study retention and user engagement with the research protocols.

Communication: Participants will receive check-in emails throughout the study reminding them of the study timeline and upcoming study assessments, other elements to anticipate, or checking in on disengaged participants to inquire about their participation status. All communication regarding treatment will be handled by Talkspace therapists within the Talkspace platform. Participants will be directed to raise any study-related questions with their therapist and/or by responding to emails from the study team or contacting the study team via phone or email.

Withdrawal of Participants: A participant may be withdrawn from the study if it is determined that they are in need of a higher level of psychiatric care than what can be provided by the study. In that case, the participant would not complete any additional study procedures. Appropriate referral and treatment would be the responsibility of the Talkspace therapist. When a participant is withdrawn from the study without their consent, the Talkspace research coordinator will directly follow up with the participant and their therapist in separate emails within 24 hours of this determination, and a copy of these communications will be sent to the UW study team for record keeping. UW coordinators will record these events in REDCap, turn off automatic surveys, and post any incentives pending delivery for the participant to be sent in the next payment cycle.

If participants choose to cease care prior to completing 12 weeks of treatment, the participant will continue to receive notifications from REDCap to complete assessments. If a participant does not complete a weekly assessment in REDCap in the first 24 hours, they will receive an automated reminder via SMS, and if in the following 24 hours they do not complete the weekly assessment, they will receive an automated reminder via email. SMS notifications for each daily survey and weekly assessment will continue to be sent throughout the 12 weeks to give participants the opportunity to complete them even if they are not responsive. If a participant reaches out to the study team via email to withdraw from the study fully, the participant will be disenrolled and no longer sent notifications via SMS and email. In addition to text and video chat services, Talkspace maintains a virtual bookshelf of articles, worksheets and educational videos therapists can share with patients.

5.2 Study Assessments

Baseline Assessments:

- Demographics
- MDE Screener
- 9-item Patient Health Questionnaire (PHQ-9)
- 8-item Neuro-Quality of Life v1.0 (Neuro-QOL) Ability to Participate in Social Roles and Activities short form
- 7-item Generalized Anxiety Scale (GAD-7)
- NIAAA Alcohol Screening Test
- IMPACT Assessment of Mania and Psychosis
- Self-reported health and health concerns
- Use of other apps and mental health treatment
- Participant Top Issue & Follow-Up Question
- Perceived Stress Scale
- Past Mental Health Treatment Experiences
- Random Assignment Level 1 Reaction Questions

Daily Assessments: Participants will be asked to answer brief, daily questions intended to capture information on daily fluctuations in mood, social and physical activity, and sleep.

Weekly Assessments:

- 9-item Patient Health Questionnaire (PHQ-9)
- 8-item Neuro-Quality of Life v1.0 (Neuro-QOL) Ability to Participate in Social Roles and Activities short form
- 7-item Generalized Anxiety Scale (GAD-7)
- Patient Global Improvement Scale

- Major Life Event & Impact on Treatment
- Participant Top Issue & Follow-Up Question
- Therapist Contact Weekly Question

Week 1: Treatment Rationale Scale

Weeks 2 and 7: Credibility Expectancy Scale (Patient's Version)

Weeks 4, 8, and 12:
- Major Life Changes
- Satisfaction with Care
- Perceived Stress scale

Weeks 4 and 10:
- Experience of Care and Health Outcomes Survey (ECHO)
- Working Alliance Inventory-Short Revised (WAI-SR)

Week 12: Exit survey.

Early exit survey: Early drop out participants will be asked to complete a 1-question early exit survey.

Talkspace data: Talkspace meta-data (number of text interactions, time texting, time between texts, number of sessions) and content data including transcripts are collected on the Talkspace platform. All identifiable information (e.g., names, usernames, links) are scrubbed from the transcripts by an automated process internal to Talkspace prior to transfer to UW. Additionally, we will collect data on treatment intensity from Talkspace's electronic record of patient contact, which consists of time spent in treatment, number of sessions/messages over 12 weeks, and treatment attendance.

Non-English-speaking or -reading: At initial contact with the study, potential participants are asked their preference about completing study procedures in either English or Spanish, which determines the language that all materials are presented in. Study treatment provided to Spanish speakers will only be provided by Spanish-speaking and/or bilingual therapists.

Interpretation: Study treatment provided to Spanish speakers will only be provided by Spanish-speaking and/or bilingual therapists. No interpreters will be utilized.

Translations: All materials will be professionally translated by Dynamic Language, a language services and translation company with extensive ties to UW. Dynamic Language follows ISO 9001 and ISO 17100 guidelines, which are internationally recognized standards for translation. Assessments with validated Spanish versions (e.g., PHQ-9) were not translated by Dynamic Language; rather, we are using the validated Spanish version.

6.0 Safety and Data Management

Risk Assessment: Some of the questions asked during assessments might make participants feel uncomfortable; however, participants may choose not to answer any question at any time and still continue with the study. There is a slight risk of loss of confidentiality, which may result in psychological or social harm (e.g., embarrassment, guilt, stress). All study staff have completed CITI/Good Clinical Practice and HIPAA training. Please refer to data security protections referenced below for additional ways that this risk is managed/reduced. We do not anticipate any risks to participants from either Talkspace, MBP, or VBP. The study will follow the guidelines in its Data and Safety Monitoring Plan.

Suicidal Ideation: Participants will complete the PHQ-9 weekly via REDCap and every three weeks within the Talkspace platform, which is standard of care for Talkspace. If the participant endorses any suicidal ideation during the PHQ-9 administration in REDCap, they will see the following information on the screen in REDCap: "If you are ever experiencing thoughts that life is not worth living, self-harm, or suicide and need to speak with someone immediately, please call 1-833-929-1721 to speak to someone 24/7, go to www.imalive.org/online to message

someone online 24/7, or call 9-1-1." Additionally, REDCap immediately and automatically alerts the Lead Investigator at Talkspace via email. Upon receipt of that email, the Lead Investigator alerts the Talkspace therapist, who will be responsible for assessing risk and engaging in safety planning. Therapists are responsible for these activities if any suicidal ideation is endorsed during the course of treatment. Therapists review completed assessments and respond to texts within 15 minutes during their office hours, and within 3 hours outside of office hours (except at night when up to 10 hours can separate responses). Talkspace has established procedures to assist therapists in executing the duties defined by their professional licensures, including incorporating Columbia University's SAFE-T protocol to assist in risk assessments and having other clinicians available for consultation. If clinical judgment suggests that the patient is at risk, patients are provided with a suicide hotline number and URL for the Crisis Text Line. The therapist follows up periodically, with frequency of follow-up determined by risk level. Assessed high-risk individuals are asked to go to the emergency room or call 911 if in immediate danger. Risk planning is undertaken with lower risk level individuals. These risk procedures are standard of care for the field.

Anticipated Direct Benefits to Participants: Participants may experience a decrease in depressive symptoms due to treatment received as part of the study.

Privacy Protections: Screening and treatment data will be stored by Talkspace on secure, encrypted servers, and data are pulled in aggregate only with randomly assigned numbers that anonymously identify patients, therapists, and virtual rooms. De-identified treatment data merged with assessment data will be given unique random identifying codes and stored on secure servers. Assessment data are stored by REDCap, which is hosted by UW.

Identifiers: Both Talkspace and UW will have access to names, email addresses, and phone numbers, which will be stored separately from study data within REDCap. Direct and indirect identifiers may be collected in content data, which the Talkspace team has access to; however, these data will be de-identified prior to sharing with the UW team.

Protected Health Information (PHI): Names, PHQ-9 scores, mental health history, and psychiatric medication information are used in the screening process within REDCap. There is an open text field asking participants to list medications they take for their mental health. Potential participants' scores on the PHQ-9, mental health history and medication information are used to screen for eligibility for the study.

Prolific Recruitment Survey: No identifiers or PHI will be gathered.

Storage of Identifiers: REDCap generates a unique user identification number for every participant, which accompanies data. This unique user identification is tied to participants' identifiable information in a single place, which is accessible only to authorized members of the study team. The Talkspace research team obtains direct and indirect identifiers by users as they appear in transcripts. UW will only obtain this data after it has been de-identified and transferred via a secure BOX folder.

Data Transfers: Treatment data from all study participants, which includes meta-data and content data, will be transferred to UW via a secure BOX account for analyses. The Talkspace team will merge the de-identified data collected and stored on REDCap (to which they already have access) to the Talkspace de-identified treatment data they collect on their platform. They then will remove the REDCap subject ID that links the REDCap data to other identifiers, generate an entirely new set of unique IDs for the merged data set, and then transfer the data to UW via a secure BOX account. These data will be stored separately from other data so that it is entirely de-identified and not linked to any identifiable information.

Meta-data include number of text interactions, time texting, and time between texts. These data are collected on the order of minutes (page views, time between sessions), and hours, days, weeks (number of texts, visits over time). Content data includes transcripts from psychotherapy sessions. All data types are routinely collected by Talkspace. As part of routine practice and as part of their terms and conditions, Talkspace explains to consumers that their information may be used for research purposes and shared with researchers in academic settings, so with patient agreement to the Talkspace Privacy Policy, the sharing of these de-identified data are allowable. Talkspace has internal processes for de-identifying data: a program called MixPanel tracks a website user using a random identifier. If and/or once a user signs up for the service, only then can Talkspace attach any identifier (e.g., email) to their website behavior, which is done retroactively by replacing MixPanel's random identifier with a new random,

numeric identifier given by the system to all user accounts. Talkspace has access to identifiable information from participants, including PHI, but all identifiable information and PHI are removed prior to transfer to UW for analyses. Data transferred for analyses will only include the random, numeric identifier. Data transferred will include dates of service, which will be transformed upon receipt by the UW team by altering all dates of service based on their relationship to a randomly selected date.

PHI for which HIPAA authorization will not be obtained: Names, assessment information, and treatment data. The minimum necessary amount of PHI to accomplish the purposes described in this protocol will be accessed, obtained and/or used. The PHI will not be reused or disclosed to any other person or entity, except as required by law, for authorized oversight of the research study, or for other research for which the use or disclosure of PHI would be permitted. The HIPAA "accounting for disclosures" requirement will be fulfilled, if applicable (see UW Medicine Compliance Policy #104). There will be reasonable safeguards to protect against identifying, directly or indirectly, any patient in any report of the research.

7.0 Secondary Use or Sharing of Information or Contact Information

Storage for Future Use: All data collected will be stored for future use. This includes all assessment data. All assessment data will be stored separately from identifiable information. Assessment data are coded with a unique study ID number, which cannot be used to identify the participant; the ID number will be stored along with the assessment data for future use. De-identified Talkspace treatment data that has been merged with assessment data will be stored for future use, and this merged data set will be coded with unique IDs that are distinct from, stored separately from, and not linked to the original separate data sets.

Data Sharing: All assessment data may be shared with other researchers. No direct identifiers will be shared. Participants consent to this sharing during the provision of informed consent. De-identified content and meta-data may also be shared with other researchers. Patients agree to this as part of Talkspace's Privacy Policy before they enter the platform and the study.

Oversight/management of Sharing: The oversight and management of this sharing will be the responsibility of project PIs.

Possible Future Uses: Possible future uses include ongoing data analysis for publication and informing future grant proposals for clinical trials.

8.0 Statistical Analysis Plan

Phase 2 is a 12-week, randomized clinical trial using a sequential multiple assignment randomized trial (SMART) design (see protocol paper[11] and incentive paper[12]). Its goals are to:

1. Test effectiveness of unlimited daily, message-based psychotherapy (MBP) for depression, compared to weekly traditionally delivered, videoconferencing-based psychotherapy (VBP). Level 1 conditions: unlimited MBP and weekly VBP.
2. Develop an evidence-based treatment sequence for those who do not respond to either MBP or VBP. Level 2 conditions for participants who do not respond to treatment: MBP+VBP_{weekly} (switch from MBP to weekly VBP with MBP), MBP+$VBP_{monthly}$ (switch from MBP to monthly VBP with MBP), VBP_{weekly}+MBP (switch from VBP to weekly VBP with MBP), and $VBP_{monthly}$+MBP (switch from VBP to monthly VBP with MBP).

Hypothesis 1: Compared to those randomized to weekly video care at baseline, patients randomized to message-based care will have greater improvements in depression (1a) and social functioning (1b), be more likely to show a treatment response (1c), and be more likely to experience remission (1d) at 5 and 12 weeks of care. Hypothesis 2: For those who fail to respond to treatment from baseline to 5 weeks, those re-randomized to receive (or retain) weekly video care will have greater improvements in depression and be more likely to show a response to treatment than those randomized to monthly video care. Hypothesis 3 (moderator): Baseline anxiety will moderate improvement in depression such that those with greater anxiety will have better response to message-based care at 5 and 12 weeks than to video-based care. Exploratory Aims: Exploratory evaluation of differences among groups in treatment engagement by week 5, ratings of therapeutic alliance at weeks 4 and 10, ratings of quality of care and amount helped by treatment at week 10, and ratings of goal satisfaction, plans to continue using treatment, and likelihood to recommend treatment to others at week 12.

<u>Sample Size and Power</u>: We propose to screen 4,789 potential participants, anticipating we will enroll 1,250 participants to arrive at a final sample of 1,000 evaluated participants, assuming a 20% dropout rate by the last assessment. In our previous psychotherapy studies, dropout rates have been on the order of 10% by final assessment. In our recent on-line studies with Google Verily, drop out has been on the order of 20%. Our large incentive arm, tested in Phase 1, is based on the incentive analyses done on the Baseline Mood Study at Verily.

<u>Level 1 Power</u>: For the quantitative primary outcome (PHQ-9) we have greater than 80% power to detect a 0.20 standard deviation across groups which corresponds to a clinically meaningful small difference. For a binary success indicator, we will consider a 50% reduction in PHQ-9 from baseline to week 5, and we have greater than 80% power to detect an absolute difference of 10% (e.g., 60% of subjects with>50% improvement versus 70% of subjects with >50% improvement).

<u>Level 2 Power</u>: The primary comparison in the second stage is the comparison of the Ultimate Plan (UP; MBP with weekly VBP) versus the Premium Plan (PP; MBP with monthly VBP) for each of the first stage randomization groups (MBP and VBP). We expect a second stage analysis sample of 324 subjects, and this corresponds to 80% power to detect a 0.32 standard deviation difference across the UP and PP groups when pooling across the primary MBP and VBP strata. Our primary second stage analysis will construct an overall UP versus PP difference stratifying on first stage randomization, and secondary analysis will consider the UP versus PP difference within the MBP and VBP groups separately.

<u>Accounting for Provider Effects</u>: Each provider will treat both MBP and VBP patients. Therefore, our primary analyses will all cluster on provider either using linear mixed models or generalized estimating equations to yield valid statistical inference. However, since treatment condition will vary within provider, the impact of provider clustering is to effectively balance or control for provider effects. Therefore, our power calculations are conservative since they do not account for the potential increase in precision associated with control for provider effects. Such within-provider randomization is in contrast to traditional cluster randomized trials where providers treat all patients in one study condition and thus increase variability associated with treatment comparisons through the well-known intra-cluster correlation coefficient.

<u>Statistical Analyses</u>: See Table 2 for analyses by hypothesis. Treatment disengagement, treatment response, and remission will be analyzed using chi-square tests and Cramér V effect sizes. 2-level linear mixed effects models using full information maximum likelihood (FIML) and an unstructured covariance matrix with client nested within clinician will be used for analyses at a single timepoint. Models predicting binary outcomes at a single timepoint will be evaluated using 2-level logistic regression models with Cholesky factorization with client nested within clinician, with Level 2 condition as the independent variable. Effect sizes for these models will be computed via pooled standard deviations of conditions at that timepoint.

Models assessing changes across time will be evaluated using 3-level mixed effects regression models with timepoint nested within client nested within clinician using FIML and an unstructured covariance matrix. Client-level effects will be allowed to randomly vary, and clinician will be a clustering variable with condition approximately balanced within clinicians. Analyses with time from baseline to week 12 will include a spline variable to account for differences in outcome trajectories from baseline to week 5 and from week 6 to week 12.

The mixed-effects models will utilize all available data. Prior research has demonstrated equivalence of longitudinal mixed model analyses using imputed versus non-imputed data (e.g., between multiple imputation and FIML).[13] Theoretically, there is no reason to expect substantial differences between FIML and MI with a reasonable sample size and complete predictors.[14] Thus, missing data will not be imputed. Cohen's *d* clinical outcome effect sizes for the model predicting PHQ-9 scores by Level 2 condition from weeks 6 to 12 will be computed as the difference between model-estimated means divided by the pooled standard deviations when the second condition was assigned at week 6. All remaining clinical outcome effect sizes will be computed as the difference between model-estimated means divided by the pooled baseline standard deviations.

We will use the Benjamini-Hochberg procedure to account for Type 1 error by adjusting for familywise error within hypothesis families (e.g., within Hypotheses 1a to 1d; within Hypothesis 2). *P* values and effect sizes with 95% confidence intervals will be reported, as appropriate.[15]

Table 2. Analyses by Hypothesis

Hypothesis	Timepoint(s)	Statistical Test	Contrast/analysis of interest
1a: Compared to those randomized to weekly video care at baseline, patients randomized to message-based care will have greater improvements in depression	Baseline to W5	3-level mixed effects model	MBP vs VBP
1b: Compared to those randomized to weekly video care at baseline, patients randomized to message-based care will have greater improvements in social functioning	Baseline to W12	3-level mixed effects model	MBP vs VBP
1c: Compared to those randomized to weekly video care at baseline, patients randomized to message-based care will be more likely to show a treatment response at 5 and 12 weeks of care.	Between W3 to W5 / W12	Chi-square tests	MBP vs VBP
1d: Compared to those randomized to weekly video care at baseline, patients randomized to message-based care will be more likely to experience remission at 5 and 12 weeks of care.	Between W3 to W5 / W12	Chi-square tests	MBP vs VBP
2: For those who fail to respond to treatment from baseline to 5 weeks, those re-randomized to receive (or retain) weekly video care will have greater improvements in depression than those randomized to monthly video care	W6 to W12	3-level mixed effects model with time centered at week 6	MBP+VBP_{weekly} vs MBP+$VBP_{monthly}$; VBP_{weekly}+MBP vs $VBP_{monthly}$+MBP
2: For those who fail to respond to treatment from baseline to 5 weeks, those re-randomized to receive (or retain) weekly video care will be more likely to show a response to treatment than those randomized to monthly video care	W12	Chi-square test	MBP+VBP_{weekly} vs MBP+$VBP_{monthly}$; VBP_{weekly}+MBP vs $VBP_{monthly}$+MBP
3 (moderator): Baseline anxiety will moderate improvement in depression such that those with greater anxiety will have better response to message-based care at 5 and 12 weeks than to video-based care.	Baseline to W12	3-level mixed effects model, with mean-centered baseline GAD-7	MBP vs VBP
Exploratory: We will explore whether there will be differences among groups in treatment engagement by week 5	Between W3 to W5	Chi-square test	MBP vs VBP
Exploratory: We will explore whether there will be differences among groups in ratings of therapeutic alliance	W4 / W10	2-level mixed effects models	W4: Level 1 conditions (MBP vs VBP)<br>W10: Level 2 conditions (MBP vs VBP; MBP+VBP_{weekly} vs MBP+$VBP_{monthly}$; VBP_{weekly}+MBP vs $VBP_{monthly}$+MBP)
Exploratory: We will explore whether there will be differences among groups in ratings of goal satisfaction, plans to continue using treatment, and likelihood to recommend treatment to others	W12	2-level logistic regression models	Level 2 conditions (MBP vs VBP; MBP+VBP_{weekly} vs MBP+$VBP_{monthly}$; VBP_{weekly}+MBP vs $VBP_{monthly}$+MBP)

Abbreviations: MBP, message-based psychotherapy; MBP+$VBP_{monthly}$, re-randomized from MBP to MBP+monthly VBP; MBP+VBP_{weekly}, re-randomized from MBP to MBP+weekly VBP; VBP, weekly video-based psychotherapy; $VBP_{monthly}$+MBP, re-randomized from weekly VBP to MBP+monthly VBP; VBP_{weekly}+MBP, re-randomized from weekly VBP to MBP+weekly VBP.

References

1. Lei H, Nahum-Shani I, Lynch K, Oslin D, Murphy SA. A “SMART” design for building individualized treatment sequences. *Annu Rev Clin Psychol*. 2012;8(1):21-48. doi:10.1146/annurev-clinpsy-032511-143152
2. Kroenke K, Spitzer RL. The PHQ-9: a new depression diagnostic and severity measure. *Psychiatr Ann*. 2002;32(9):509-515. doi:10.3928/0048-5713-20020901-06
3. Busner J, Targum SD. The Clinical Global Impressions Scale: applying a research tool in clinical practice. *Psychiatry (Edgmont)*. 2007;4(7):28-37.
4. Harris PA, Taylor R, Minor BL, et al. The REDCap consortium: building an international community of software platform partners. *J Biomed Inform*. 2019;95:103208. doi:10.1016/j.jbi.2019.103208
5. Harris PA, Taylor R, Thielke R, Payne J, Gonzalez N, Conde JG. Research electronic data capture (REDCap)—a metadata-driven methodology and workflow process for providing translational research informatics support. *J Biomed Inform*. 2009;42(2):377-381. doi:10.1016/j.jbi.2008.08.010
6. Teitcher JE, Bockting WO, Bauermeister JA, Hoefer CJ, Miner MH, Klitzman RL. Detecting, preventing, and responding to "fraudsters" in internet research: ethics and tradeoffs. *J Law Med Ethics*. 2015;43(1):116-133. doi:10.1111/jlme.12200
7. Pozzar R, Hammer MJ, Underhill-Blazey M, et al. Threats of bots and other bad actors to data quality following research participant recruitment through social media: cross-sectional questionnaire. *J Med Internet Res*. 2020;22(10):e23021. doi:10.2196/23021
8. Campbell CK, Ndukwe S, Dubé K, Sauceda JA, Saberi P. Overcoming challenges of online research: measures to ensure enrollment of eligible participants. *J Acquir Immune Defic Syndr*. 2022;91(2):232-236. doi:10.1097/QAI.0000000000003035
9. Storozuk A, Ashley M, Delage V, Maloney EA. Got bots? Practical recommendations to protect online survey data from bot attacks. *Quant Meth Psychol*. 2020;16(5):472-481. doi:10.20982/tqmp.16.5.p472
10. Spitzer RL, Kroenke K, Williams JBW, Löwe B. A brief measure for assessing generalized anxiety disorder: the GAD-7. *Arch Intern Med*. 2006;166(10):1092-1097. doi:10.1001/archinte.166.10.1092
11. Areán P, Hull D, Pullmann MD, Heagerty PJ. Protocol for a sequential, multiple assignment, randomised trial to test the effectiveness of message-based psychotherapy for depression compared with telepsychotherapy. *BMJ Open*. 2021;11(11):e046958. doi:10.1136/bmjopen-2020-046958
12. Griffith Fillipo IR, Pullmann MD, Hull TD, et al. Participant retention in a fully remote trial of digital psychotherapy: comparison of incentive types. *Front Digit Health*. 2022;4:963741. doi:10.3389/fdgth.2022.963741
13. Twisk J, de Boer M, de Vente W, Heymans M. Multiple imputation of missing values was not necessary before performing a longitudinal mixed-model analysis. *J Clin Epidemiol*. 2013;66(9):1022-1028. doi:10.1016/j.jclinepi.2013.03.017
14. Graham JW, Olchowski AE, Gilreath TD. How many imputations are really needed? Some practical clarifications of multiple imputation theory. *Prev Sci*. 2007;8(3):206-213. doi:10.1007/s11121-007-0070-9
15. Harrington D, D'Agostino RB Sr, Gatsonis C, et al. New guidelines for statistical reporting in the journal. *N Engl J Med*. 2019;381(3):285-286. doi:10.1056/NEJMe1906559
