# Supplementary material for: Message-Based vs Video-Based Psychotherapy for Depression: A Randomized Clinical Trial
Source: JAMA Netw Open. 2025 Oct 30;8(10):e2540065. doi: 10.1001/jamanetworkopen.2025.40065 (PMC12576495; doi:10.1001/jamanetworkopen.2025.40065)
Supplement: Supplement 2. — eMethods 1. Identification and Management of Suspected Fraudulent Participation eMethods 2. Data Management Statement eMethods 3. Intention-to-Treat Procedures eMethods 4. Supplemental Measure Descriptions eMethods 5. Coding of Therapist Value eTable 1. Parameter Estimates of Multilevel Model Predicting Change in Neuro-QOL Scores From Baseline to Week 12 by First Condition eTable 2. Parameter Estimates of Multilevel Model Predicting Change in WAI-SR Scores From Week 4 to Week 10 by Second Condition eFigure. WAI-SR Model-Estimated Means From Week 4 to Week 10 by Second Condition eTable 3. Parameter Estimates of Multilevel Models Predicting Ratings on Quality of Care and Amount Helped at Week 10 by Second Condition eTable 4. Parameter Estimates of Multilevel Mixed Effects Logistic Regression Model Predicting Goal Satisfaction at Week 12 by Second Condition eTable 5. Parameter Estimates of Multilevel Mixed Effects Logistic Regression Model Predicting Plans to Continue Using Treatment at Week 12 by Second Condition eTable 6. Parameter Estimates of Multilevel Mixed Effects Logistic Regression Model Predicting Recommendation of Treatment to Others at Week 12 by Second Condition eReferences. [file jamanetwopen-e2540065-s002.pdf]

Supplementary Online Content

Pullmann MD, Rouvere J, Raue PJ, et al. Message-based vs video-based psychotherapy for depression: a randomized clinical trial. *JAMA Netw Open.* 2025;8(10):e2540065. doi:10.1001/jamanetworkopen.2025.40065

eMethods 1. Identification and Management of Suspected Fraudulent Participation

eMethods 2. Data Management Statement

eMethods 3. Intention-to-Treat Procedures

eMethods 4. Supplemental Measure Descriptions

eMethods 5. Coding of Therapist Value

eTable 1. Parameter Estimates of Multilevel Model Predicting Change in Neuro-QOL Scores From Baseline to Week 12 by First Condition

eTable 2. Parameter Estimates of Multilevel Model Predicting Change in WAI-SR Scores From Week 4 to Week 10 by Second Condition

eFigure. WAI-SR Model-Estimated Means From Week 4 to Week 10 by Second Condition

eTable 3. Parameter Estimates of Multilevel Models Predicting Ratings on Quality of Care and Amount Helped at Week 10 by Second Condition

eTable 4. Parameter Estimates of Multilevel Mixed Effects Logistic Regression Model Predicting Goal Satisfaction at Week 12 by Second Condition

eTable 5. Parameter Estimates of Multilevel Mixed Effects Logistic Regression Model Predicting Plans to Continue Using Treatment at Week 12 by Second Condition

eTable 6. Parameter Estimates of Multilevel Mixed Effects Logistic Regression Model Predicting Recommendation of Treatment to Others at Week 12 by Second Condition

eReferences.

This supplementary material has been provided by the authors to give readers additional information about their work.

eMethods 1. Identification and Management of Suspected Fraudulent Participation

To avoid fraudulent participation and the collection of data inconsistent with the target population, we implemented preventive and reactive methods pre, during, and post data collection. Respondents who gave responses in screening and baseline surveys that we identified as suspicious were removed prior to enrollment and randomization. These instances included stating an age that contradicted their self-reported date of birth, providing inconsistent information around their identity, and submission of multiple surveys within a close time frame with extremely similar or identical responses. Fraudulent participation was also identified in enrolled participants during and post data collection through suspicious and inconsistent responses. Depending on when they were identified, these participants were removed from the study or had their data removed from analyses.

© 2025 Pullmann MD et al. *JAMA Network Open.*

eMethods 2. Data Management Statement

All data are encrypted, time-stamped, de-identified, and stored in a secure server at the University of Washington.

© 2025 Pullmann MD et al. *JAMA Network Open.*

eMethods 3. Intention-to-Treat Procedures

As an intention-to-treat study, participants who dropped out of treatment continued to receive surveys, and those who stopped completing surveys were permitted to remain in treatment. Participants who were not randomized to a second condition due to missing self-reported and therapist-reported measures prior to week 6 were permitted to continue treatment in their baseline assignment.

© 2025 Pullmann MD et al. *JAMA Network Open.*

eMethods 4. Supplemental Measure Descriptions

Demographics

Demographic data were collected via a measure that has been applied in other online clinical and survey studies to request demographic information from participants. Participants self-reported gender, age, race, ethnicity, income categories, and education at baseline.

Working Alliance Inventory-Short Revised

Participants rated therapeutic alliance using the Working Alliance Inventory-Short Revised (WAI-SR),[1] a 12-item questionnaire evaluating three domains: goals, tasks, and bond. Items are rated on a scale from 1 to 5. Total scores range from 12 to 60, with higher scores indicating a more effective therapeutic relationship.

Experience of Care and Health Outcomes Survey

Participants rated quality of care via an adapted version of item 28 of the Consumer Assessment of Healthcare Providers and Systems (CAHPS) Experience of Care and Health Outcomes (ECHO) survey[2]: "In the past 4 weeks, using any number from 0 to 10, where 0 is the worst mental health care possible and 10 is the best mental health care possible, what number would you use to rate the mental health care you received from Talkspace?"

Generalized Anxiety Disorder-7

The Generalized Anxiety Disorder-7 (GAD-7)[3,4] is a 7-item assessment for generalized anxiety and has been validated as an online instrument. Respondents are asked to rate their experience of relevant symptoms in the last two weeks on a scale of 0-3, for a total score ranging from 0-21. Higher scores indicate more severe anxiety symptoms.

Clinical Global Impressions-Improvement Scale

The Clinical Global Impressions-Improvement Scale (CGI-I)[5] is a one-item clinician-rated assessment of the amount a patient has improved compared to baseline. At week 5, clinicians were asked to rate their perception of their client's change in condition since the beginning of treatment. The query is rated on a 7-point scale, with scores of: 1 (very much improved), 2 (much improved), 3 (minimally improved), 4 (no change), 5 (minimally worse), 6 (much worse), and 7 (very much worse).

© 2025 Pullmann MD et al. *JAMA Network Open.*

eMethods 5. Coding of Therapist Value

Of the 60 of 850 (7.1%) participants who switched therapists, therapist value was coded as the longest-used therapist (n = 37), most recent therapist due to missing therapist switch dates (n = 7), first therapist due to switch to a nonstudy therapist (n = 14), and first therapist due to no available in-state therapists after the original therapist dropped from the study (n = 2).

eTable 1. Parameter Estimates of Multilevel Model Predicting Change in Neuro-QOL Scores From Baseline to Week 12 by First Condition

Predictors	*b* (SE)	*P* value	Mean (SE)[a] at baseline	Mean (SE) at week 5	Mean (SE) at week 12
Intercept (VBP)	39.68 (0.21)	**<.001**	39.68 (0.21)	41.37 (0.24)	42.79 (0.35)
Time	0.34 (0.04)	**<.001**	NA	NA	NA
Spline	-0.16 (0.06)	**.008**	NA	NA	NA
MBP	-0.23 (0.28)	.41	39.45 (0.21)	41.37 (0.24)	43.35 (0.34)
MBP x Time	0.05 (0.06)	.42	NA	NA	NA
MBP x Spline	0.04 (0.08)	.61	NA	NA	NA

Abbreviations: MBP, message-based psychotherapy; NA, not applicable; Neuro-QOL, Neuro-Quality of Life v1.0 Ability to Participate in Social Roles and Activities short form; SE, standard error; VBP, weekly video-based psychotherapy.
[a]Model-estimated means and standard errors.

© 2025 Pullmann MD et al. *JAMA Network Open.*

eTable 2. Parameter Estimates of Multilevel Model Predicting Change in WAI-SR Scores From Week 4 to Week 10 by Second Condition

Predictors	*b* (SE)	*P* value	Mean (SE)[a] at week 4	Mean (SE) at week 10
Intercept (VBP)	46.88 (1.43)	**<.001**	46.88 (1.43)	49.29 (1.44)
Time[b]	2.41 (1.80)	.19	NA	NA
MBP	-1.99 (1.74)	.25	44.90 (1.31)	46.81 (1.35)
MBP + weekly VBP	-13.97 (1.66)	**<.001**	32.91 (1.20)	37.66 (1.22)
MBP + monthly VBP	-12.92 (1.74)	**<.001**	33.97 (1.32)	37.98 (1.33)
Weekly VBP + MBP	-6.39 (1.73)	**<.001**	40.50 (1.30)	41.00 (1.31)
Monthly VBP + MBP	-6.60 (1.71)	**<.001**	40.29 (1.27)	41.58 (1.28)
MBP x Time	-0.50 (2.45)	.84	NA	NA
MBP + weekly VBP x Time	2.35 (2.32)	.31	NA	NA
MBP + monthly VBP x Time	1.61 (2.43)	.51	NA	NA
Weekly VBP + MBP x Time	-1.90 (2.42)	.43	NA	NA
Monthly VBP + MBP x Time	-1.11 (2.39)	.64	NA	NA

Abbreviations: MBP, message-based psychotherapy; MBP + monthly VBP, rerandomized from MBP to MBP + monthly VBP; MBP + weekly VBP, rerandomized from MBP to MBP + weekly VBP; NA, not applicable; SE, standard error; VBP, weekly video-based psychotherapy; monthly VBP + MBP, rerandomized from weekly VBP to MBP + monthly VBP; weekly VBP + MBP, rerandomized from weekly VBP to MBP + weekly VBP; WAI-SR, Working Alliance Inventory-Short Revised.
[a]Model-estimated means and standard errors.
[b]Time entered as a categorical variable; reference: week 4.

© 2025 Pullmann MD et al. *JAMA Network Open.*

eFigure. WAI-SR Model-Estimated Means From Week 4 to Week 10 by Second Condition

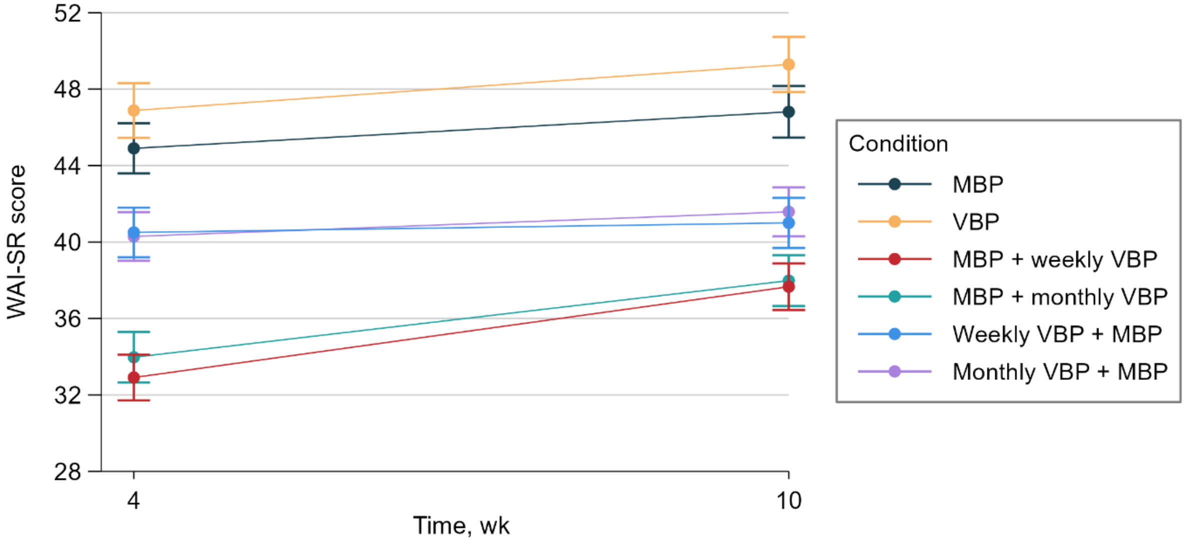

Data points represent estimated means and error bars, SEs. MBP, message-based psychotherapy; MBP + monthly VBP, rerandomized from MBP to MBP + monthly VBP; MBP + weekly VBP, rerandomized from MBP to MBP + weekly VBP; VBP, weekly video-based psychotherapy; monthly VBP + MBP, rerandomized from weekly VBP to MBP + monthly VBP; weekly VBP + MBP, rerandomized from weekly VBP to MBP + weekly VBP; WAI-SR, Working Alliance Inventory-Short Revised.

© 2025 Pullmann MD et al. *JAMA Network Open.*

eTable 3. Parameter Estimates of Multilevel Models Predicting Ratings on Quality of Care and Amount Helped at Week 10 by Second Condition

	Quality of Care			Amount Helped		
**Predictors**	***b* (SE)**	***P* value**	**Mean (SE)[a]**	***b* (SE)**	***P* value**	**Mean (SE)**
Intercept (VBP)	8.02 (0.27)	**<.001**	8.02 (0.27)	2.62 (0.11)	**<.001**	2.62 (0.11)
MBP	-0.59 (0.35)	.09	7.43 (0.25)	-0.20 (0.14)	.15	2.42 (0.10)
MBP + weekly VBP	-1.90 (0.33)	**<.001**	6.11 (0.23)	-0.71 (0.13)	**<.001**	1.90 (0.09)
MBP + monthly VBP	-2.21 (0.34)	**<.001**	5.80 (0.25)	-0.84 (0.14)	**<.001**	1.78 (0.10)
Weekly VBP + MBP	-1.63 (0.34)	**<.001**	6.38 (0.24)	-0.60 (0.14)	**<.001**	2.01 (0.10)
Monthly VBP + MBP	-1.82 (0.34)	**<.001**	6.20 (0.24)	-0.74 (0.14)	**<.001**	1.88 (0.10)

Abbreviations: MBP, message-based psychotherapy; MBP + monthly VBP, rerandomized from MBP to MBP + monthly VBP; MBP + weekly VBP, rerandomized from MBP to MBP + weekly VBP; SE, standard error; VBP, weekly video-based psychotherapy; monthly VBP + MBP, rerandomized from weekly VBP to MBP + monthly VBP; weekly VBP + MBP, rerandomized from weekly VBP to MBP + weekly VBP.
[a]Model-estimated means and standard errors.

© 2025 Pullmann MD et al. *JAMA Network Open.*

eTable 4. Parameter Estimates of Multilevel Mixed Effects Logistic Regression Model Predicting Goal Satisfaction at Week 12 by Second Condition

**Predictors**	***b* (SE)**	***P* value**	**OR (95% CI)**
Intercept (VBP)	2.08 (0.39)	**<.001**	NA
MBP	-0.31 (0.50)	.54	0.73 (0.28-1.96)
MBP + weekly VBP	-1.77 (0.44)	**<.001**	0.17 (0.07-0.40)
MBP + monthly VBP	-2.05 (0.44)	**<.001**	0.13 (0.05-0.31)
Weekly VBP + MBP	-1.53 (0.45)	**<.001**	0.22 (0.09-0.52)
Monthly VBP + MBP	-1.75 (0.44)	**<.001**	0.17 (0.07-0.41)

Abbreviations: CI, confidence interval; MBP, message-based psychotherapy; MBP + monthly VBP, rerandomized from MBP to MBP + monthly VBP; MBP + weekly VBP, rerandomized from MBP to MBP + weekly VBP; NA, not applicable; OR, odds ratio; SE, standard error; VBP, weekly video-based psychotherapy; monthly VBP + MBP, rerandomized from weekly VBP to MBP + monthly VBP; weekly VBP + MBP, rerandomized from weekly VBP to MBP + weekly VBP.

© 2025 Pullmann MD et al. *JAMA Network Open.*

eTable 5. Parameter Estimates of Multilevel Mixed Effects Logistic Regression Model Predicting Plans to Continue Using Treatment at Week 12 by Second Condition

Predictors	*b* (SE)	*P* value	OR (95% CI)
Intercept (VBP)	0.60 (0.25)	**.02**	NA
MBP	-0.50 (0.33)	.14	0.61 (0.32-1.18)
MBP + weekly VBP	-1.01 (0.32)	**.002**	0.37 (0.20-0.68)
MBP + monthly VBP	-1.17 (0.33)	**<.001**	0.31 (0.16-0.60)
Weekly VBP + MBP	-0.78 (0.33)	**.02**	0.46 (0.24-0.88)
Monthly VBP + MBP	-0.65 (0.32)	**.04**	0.52 (0.28-0.98)

Abbreviations: CI, confidence interval; MBP, message-based psychotherapy; MBP + monthly VBP, rerandomized from MBP to MBP + monthly VBP; MBP + weekly VBP, rerandomized from MBP to MBP + weekly VBP; NA, not applicable; OR, odds ratio; SE, standard error; VBP, weekly video-based psychotherapy; monthly VBP + MBP, rerandomized from weekly VBP to MBP + monthly VBP; weekly VBP + MBP, rerandomized from weekly VBP to MBP + weekly VBP.

© 2025 Pullmann MD et al. *JAMA Network Open.*

eTable 6. Parameter Estimates of Multilevel Mixed Effects Logistic Regression Model Predicting Recommendation of Treatment to Others at Week 12 by Second Condition

**Predictors**	***b* (SE)**	***P* value**	**OR (95% CI)**
Intercept (VBP)	3.64 (0.73)	**<.001**	NA
MBP	-1.70 (0.80)	**.03**	0.18 (0.04-0.88)
MBP + weekly VBP	-2.71 (0.76)	**<.001**	0.07 (0.02-0.29)
MBP + monthly VBP	-3.37 (0.76)	**<.001**	0.03 (0.01-0.15)
Weekly VBP + MBP	-2.04 (0.78)	**.009**	0.13 (0.03-0.60)
Monthly VBP + MBP	-2.51 (0.76)	**.001**	0.08 (0.02-0.36)

Abbreviations: CI, confidence interval; MBP, message-based psychotherapy; MBP + monthly VBP, rerandomized from MBP to MBP + monthly VBP; MBP + weekly VBP, rerandomized from MBP to MBP + weekly VBP; NA, not applicable; OR, odds ratio; SE, standard error; VBP, weekly video-based psychotherapy; monthly VBP + MBP, rerandomized from weekly VBP to MBP + monthly VBP; weekly VBP + MBP, rerandomized from weekly VBP to MBP + weekly VBP.

© 2025 Pullmann MD et al. *JAMA Network Open.*

eReferences.

1. Hatcher RL, Gillaspy JA. Development and validation of a revised short version of the working alliance inventory. *Psychother Res*. 2006;16(1):12-25. doi:10.1080/10503300500352500
2. Daniels AS, Shaul JA, Greenberg P, Cleary PD. The Experience of Care and Health Outcomes Survey (ECHO): a consumer survey to collect ratings of behavioral health care treatment, outcomes and plans. In: The Use of Psychological Testing for Treatment Planning and Outcomes Assessment: Instruments for Adults. Vol 3. 3rd ed. Lawrence Erlbaum Associates Publishers; 2004:839-866.
3. Spitzer RL, Kroenke K, Williams JBW, Löwe B. A brief measure for assessing generalized anxiety disorder: the GAD-7. *Arch Intern Med*. 2006;166(10):1092-1097. doi:10.1001/archinte.166.10.1092
4. Johnson SU, Ulvenes PG, Øktedalen T, Hoffart A. Psychometric properties of the General Anxiety Disorder 7-Item (GAD-7) scale in a heterogeneous psychiatric sample. *Front Psychol*. 2019;10:1713. doi:10.3389/fpsyg.2019.01713
5. Busner J, Targum SD. The Clinical Global Impressions Scale: applying a research tool in clinical practice. *Psychiatry (Edgmont)*. 2007;4(7):28-37.

© 2025 Pullmann MD et al. *JAMA Network Open.*
