# Supplementary material for: Message-Based vs Video-Based Psychotherapy for Depression: A Randomized Clinical Trial
Source: JAMA Netw Open. 2025 Oct 30;8(10):e2540065. doi: 10.1001/jamanetworkopen.2025.40065 (PMC12576495; doi:10.1001/jamanetworkopen.2025.40065)
Supplement: Supplement 3. — Data Sharing Statement [file jamanetwopen-e2540065-s003.pdf]

Data Sharing Statement

Pullmann. Message-Based vs Video-Based Psychotherapy for Depression. *JAMA Netw Open*. Published October 30, 2025. doi:10.1001/jamanetworkopen.2025.40065

Data
Additional Information: ClinicalTrials.gov Identifier NCT04513080
Data available: Yes
Data types: Deidentified participant data
How to access data: praue@uw.edu
When available: With publication

Supporting Documents
Document types: None

Additional Information
Who can access the data: Researchers whose proposed use of the data has been approved
Types of analyses: For a specified purpose
Mechanisms of data availability: With investigator support
